# Persistent organic pollutants and β-cell toxicity: a comprehensive review

**DOI:** 10.1152/ajpendo.00358.2021

**Published:** 2022-02-14

**Authors:** Myriam P. Hoyeck, Geronimo Matteo, Erin M. MacFarlane, Ineli Perera, Jennifer E. Bruin

**Affiliations:** ^1^Department of Biology and Institute of Biochemistry, Carleton University, Ottawa, Ontario, Canada; ^2^Department of Biology, University of Ottawa, Ottawa, Ontario, Canada; ^3^Environmental Health Science and Research Bureau, Health Canada, Ottawa, Ontario, Canada

**Keywords:** diabetes, hyperglycemia, insulin, persistent organic pollutants, β-cell dysfunction

## Abstract

Persistent organic pollutants (POPs) are a diverse family of contaminants that show widespread global dispersion and bioaccumulation. Humans are continuously exposed to POPs through diet, air particles, and household and commercial products; POPs are consistently detected in human tissues, including the pancreas. Epidemiological studies show a modest but consistent correlation between exposure to POPs and increased diabetes risk. The goal of this review is to provide an overview of epidemiological evidence and an in-depth evaluation of the in vivo and in vitro evidence that POPs cause β-cell toxicity. We review evidence for six classes of POPs: dioxins, polychlorinated biphenyls (PCBs), organochlorine pesticides (OCPs), organophosphate pesticides (OPPs), flame retardants, and per- and polyfluoroalkyl substances (PFAS). The available data provide convincing evidence implicating POPs as a contributing factor driving impaired glucose homeostasis, β-cell dysfunction, and altered metabolic and oxidative stress pathways in islets. These findings emphasize the need to consider the endocrine pancreas in toxicity assessments. Our review also highlights significant gaps in the literature assessing islet-specific endpoints after both in vivo and in vitro POP exposure. In addition, most rodent studies do not consider the impact of biological sex or secondary metabolic stressors in mediating the effects of POPs on glucose homeostasis and β-cell function. We discuss key gaps and limitations that should be assessed in future studies.

## Graphical abstract

**Figure:**
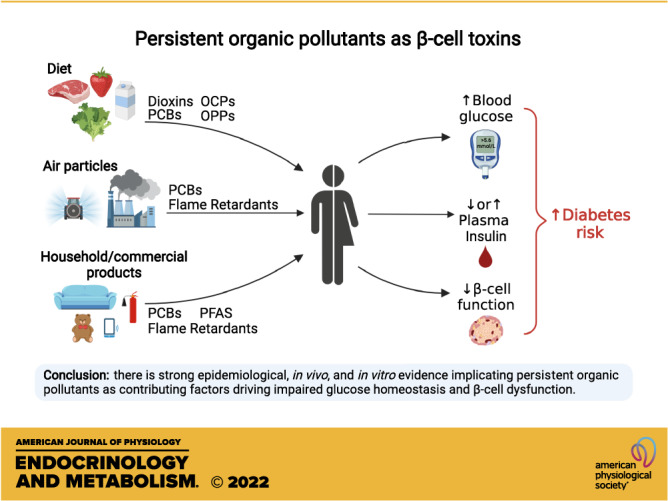

## INTRODUCTION

Type 2 diabetes (T2D) prevalence is rapidly increasing, with >460 million people living with diabetes worldwide (1). T2D is characterized by chronic hyperglycemia caused by systemic insulin resistance, impaired insulin secretion by pancreatic β-cells, and/or reduced β-cell mass (2). The islets of Langerhans comprise the endocrine portion of the pancreas, consisting of five endocrine cell types that work synergistically to regulate blood glucose (3). Insulin-secreting β-cells are the most predominant cell type in islets and are tightly regulated by stimuli such as glucose, amino acids, fatty acids, and hormones (3). Pancreatic β-cells are formed during embryonic development and are generally nonproliferative, except during periods of metabolic stress (4); thus, damage to β-cells can have long-term metabolic consequences.

Genome-wide association studies have shown that most T2D risk loci are involved in regulating insulin secretion (5–7), suggesting a critical role for β-cells in driving diabetes risk. However, rising diabetes rates cannot be explained by genetics alone, and there are clearly additional environmental factors influencing diabetes pathogenesis (7–9). Although inactive lifestyle and poor diet are important contributors to the development of T2D (9), an increasing number of studies are reporting a link between chronic exposure to environmental pollutants and T2D risk (10–14). Our review focuses on persistent organic pollutants (POPs), as the relationship between POPs and T2D risk is particularly consistent (14–19).

POPs are a diverse family of environmental contaminants that resist degradation, leading to widespread global dispersion and bioaccumulation (20–22). POPs are detected in the pancreas of humans (23, 24) and rodents (25–27) and are eliminated from the pancreas more slowly than from other tissues (25, 26), suggesting that the pancreas is a reservoir for POP accumulation. Our laboratory has also shown that systemic exposure of mice to POPs activates xenobiotic metabolism enzymes in the endocrine pancreas, confirming that islets are directly exposed to POPs in vivo (25, 28, 29). Given the critical role of islets in maintaining glucose homeostasis and the limited regenerative capacity of β-cells, it is of utmost importance to identify environmental pollutants that disrupt β-cell health and function. The goal of this review is to provide an in-depth evaluation of the evidence that POPs cause β-cell toxicity.

## REVIEW OVERVIEW

**Figure 1.**
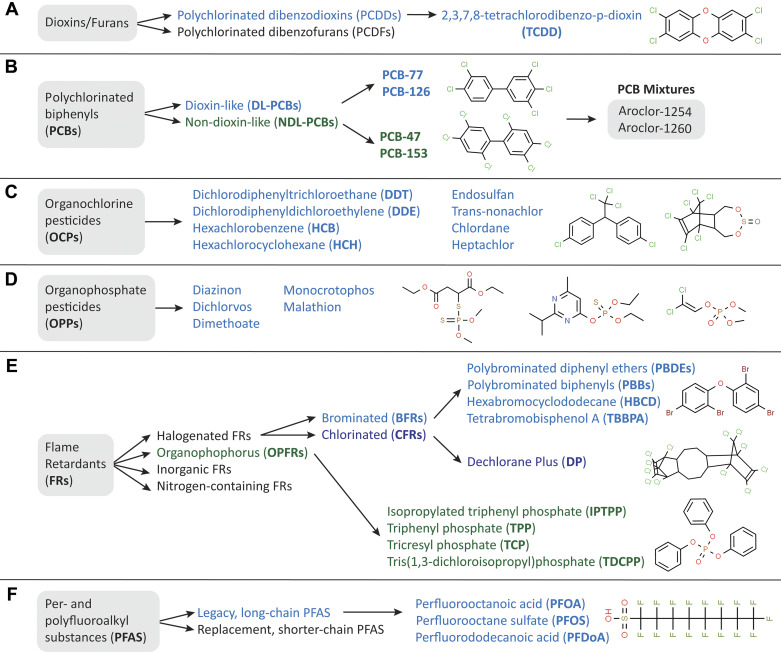
Overview of persistent organic pollutant (POP) subclasses. This review focuses on 6 classes of POPs: dioxins/furans (*A*), polychlorinated biphenyls (PCBs; *B*), organochlorine pesticides (OCPs; *C*), organophosphate pesticides (OPPs; *D*), flame retardants (*E*), and per- and polyfluoroalkyl substances (PFAS; *F*).

This review explores six classes of POPs: *1*) dioxins, *2*) polychlorinated biphenyls (PCBs), *3*) organochlorine pesticides (OCPs), *4*) organophosphate pesticides (OPPs), *5*) flame retardants, and *6*) per- and polyfluoroalkyl substances (PFAS) (Fig. 1). Within each class of POPs, we review epidemiological, in vivo, and in vitro studies that assessed glucose homeostasis, islet function and biochemical characteristics, and β-cell stress and/or viability endpoints. We focus on adult exposure to POPs, as developmental exposure is beyond the scope of this review. We made a significant effort to consider all studies that met these criteria but acknowledge that some publications may have been unintentionally missed.

**Figure 2.**
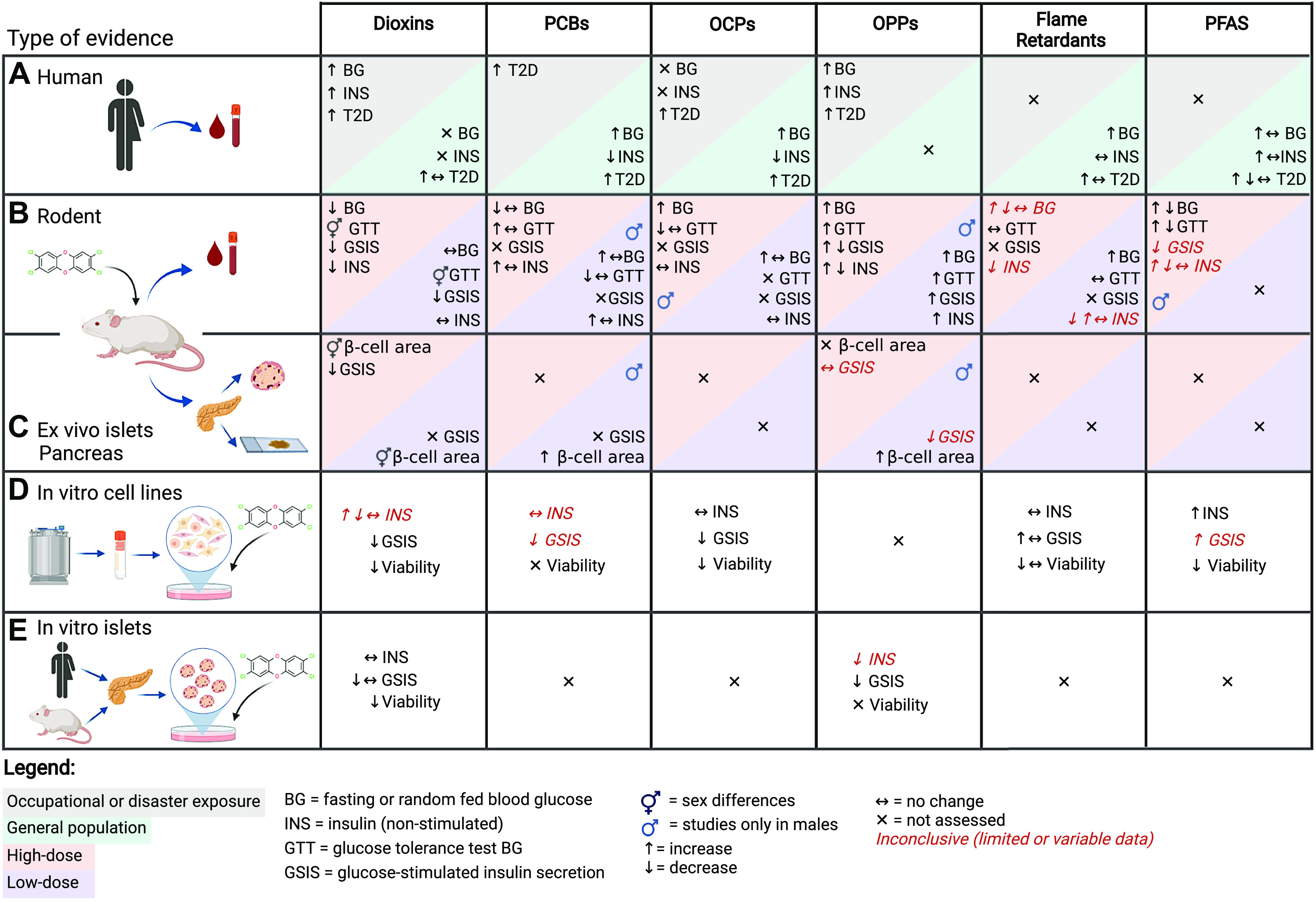
Summary of the evidence linking persistent organic pollutant (POP) exposure with impaired glucose homeostasis and β-cell dysfunction. The data provided include evidence from epidemiology studies in human populations (*A*), rodent studies with metabolic assessments after in vivo chemical exposure (*B*), analysis of isolated islets ex vivo and pancreas tissue collected from rodents after in vivo chemical exposure (*C*), immortalized β-cell lines exposed directly to chemicals in vitro (*D*), and isolated primary rodent or human islets exposed directly to chemicals *in vitro (E*). Data summarized for dioxins, polychlorinated biphenyls (PCBs), organochlorine pesticides (OCPs), organophosphate pesticides (OPPs), flame retardants, and per- and polyfluoroalkyl substances (PFAS). Created with BioRender.com.

We first present epidemiological evidence to broadly summarize the link between each class of POPs and T2D risk in humans (Fig. 2*A*). We classify populations subjected to occupational or disaster-type exposure as high dose and exposure in the general population as low dose. We also present circulating POP concentrations to provide context for the general level of exposure in humans. When possible, we highlight any evidence pertaining to the relationship between circulating POP concentrations and plasma insulin levels, although this was generally limited. While epidemiological data cannot be used to infer causation, these data provide helpful context for experimental studies in model systems.

We next consider the causal relationship between POP exposure and β-cell toxicity based on rodent models in vivo (Fig. 2, *B* and *C*). We summarize the effect of POP administration on glucose homeostasis and plasma insulin levels in vivo as an indication of β-cell function (Fig. 2*B*). However, the strongest evidence for β-cell toxicity comes from in vivo POP exposure studies where islets were isolated for evaluation of glucose-stimulated insulin secretion (GSIS) ex vivo or molecular-level effects, and pancreas was harvested for histological analysis of islets (Fig. 2*C*).

To interpret rodent studies, it is important to consider how the chemical administration protocol compares to human exposure. We classified timing of exposure as acute (<48 h), short term (2–14 days), prolonged (2–12 wk), or chronic (>12 wk); acute/short-term models provide insight into the immediate toxic effects of chemicals, whereas prolonged/chronic models are used to assess long-term effects due to accumulation of chemicals within an organism. It was more difficult to classify dosage, because many studies do not measure circulating POP concentrations, but we attempted to distinguish between supraphysiological high-dose protocols that exceed general human exposure and physiologically relevant protocols. Although supraphysiological dosing protocols do not mimic human exposure, these studies provide insight into mechanism of action and thus are included in this review. We further classified physiologically relevant exposure protocols as high dose that mimics occupational or disaster-type exposure and low dose that reflects the general population.

There are numerous different cell culture models used to study the direct effects of POP exposure on β-cell physiology in vitro (Fig. 2, *D* and *E*). We consider both the strength of the model and the chemical exposure protocol in evaluating the evidence. In vitro models include immortalized rodent β-cell lines (Fig. 2*D*), engineered human β-cell lines, primary rodent and human islets (Fig. 2*E*), and human stem cell-derived pancreatic endocrine cells. Immortalized rodent cell lines are useful for toxicity testing but limited in their capacity to accurately model human β-cell physiology (30–32). Isolated primary rodent islets are a better model for β-cell physiology and frequently used in toxicology but are architecturally different from human islets (33–35). Primary human islets from deceased organ donors are an excellent resource for studying β-cells (36), but access and scalability for pollutant exposure studies are limited. Human pluripotent stem cell-derived insulin-secreting β-like cells are more broadly accessible and highly scalable, although they are only recently being explored as a model for β-cell toxicity (37, 38) and thus are not considered in this review. In summary, we present evidence from diverse in vitro models exploring the direct effects of POPs on β-cells but weigh studies in primary islets (Fig. 2*E*) more heavily than those in immortalized rodent cell lines (Fig. 2*D*).

This review aims to identify POPs that disrupt β-cell function either in vivo or in vitro, with GSIS being considered the gold standard outcome measure. However, insulin secretion was often assessed with unconventional approaches, which we highlight for clarity of interpretation (Figs. 4–11). For this review, we define “conventional static GSIS” as a 1-h incubation in 2.8 mM low-glucose Krebs-Ringer bicarbonate buffer (KRBB) followed by a 1-h incubation in 16.7 mM high-glucose KRBB or simultaneous 1-h incubation in 2.8 mM low-glucose KRBB or 16.7 mM high-glucose KRBB on different islet aliquots.

## DIOXINS

### Overview of Dioxins and Human Exposure

Dioxins are a class of polyhalogenated POPs that include polychlorinated dibenzodioxins (PCDDs) and polychlorinated dibenzofurans (PCDFs, Fig. 1*A*) (39). These compounds are formed as by-products of combustion processes such as waste incineration and chemical manufacturing (e.g., herbicides). Dioxins are extremely resistant to biological degradation and permeate the environment globally (39). The most toxic and widely studied PCDD is 2,3,7,8-tetrachlorodibenzo-*p*-dioxin (TCDD, referred to simply as “dioxin”, Fig. 1*A*), with a half-life of 3–9 yr in adults (40–42).

**Figure 3.**
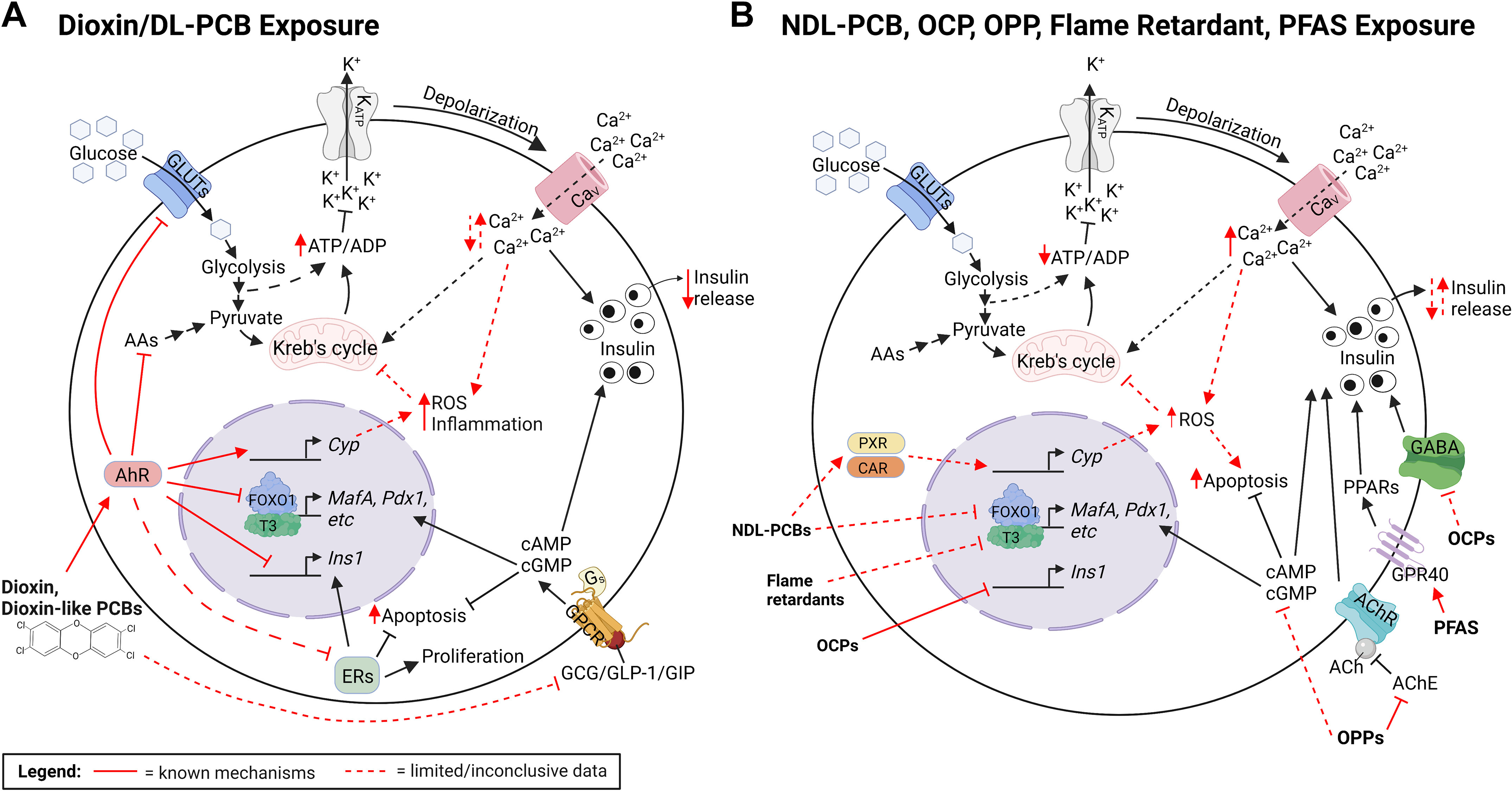
Proposed mechanisms for persistent organic pollutant (POP)-induced β-cell toxicity. This figure summarizes key mechanisms through which dioxins, polychlorinated biphenyls (PCBs), organochlorine pesticides (OCPs), organophosphate pesticides (OPPs), flame retardants, and per- and polyfluoroalkyl substances (PFAS) may alter β-cell function. Solid red arrows indicate well-established modes of action, whereas dashed red arrows indicate proposed mechanistic effects. *A*: observed changes in β-cell physiology following exposure to dioxin and dioxin-like (DL)-PCBs, including increased ATP/ADP, intracellular Ca^2+^, and reactive oxygen species (ROS) levels leading to impaired insulin secretion; these effects are predicted to be mediated by aryl hydrocarbon receptor (AhR) activation. *B*: observed effects of non-dioxin-like (NDL)-PCBs, OCPs, OPPs, flame retardants, and PFAS on β-cell physiology, including decreased ATP/ADP, increased intracellular Ca^2+^, and impaired insulin secretion; these effects are proposed to be mediated by pregnane X receptor (PXR)/constitutive aldosterone receptor (CAR), gamma aminobutyric acid (GABA) receptor, acetylcholinesterase (AChE), forkhead box protein O1 (FOXO1)/thyroid hormone (triiodothyronine, T_3_), and free fatty acid receptor 1 (GPR40), respectively. AAs, amino acids; ACh, acetylcholine; AChR, acetylcholine receptor; cAMP, cyclic adenosine monophosphate; cGMP, cyclic guanosine monophosphate; CYP, cytochrome *P*-450; ERs, estrogen receptors; GCG, glucagon; GIP, gastric inhibitory peptide; GLP-1, glucagon-like peptide-1; GPCR, G protein-coupled receptor; INS1, insulin 1; MAFA, MAF BZIP transcription factor A; PDX1, insulin promoter factor 1; PPARs, Peroxisome proliferator-activated receptors. Created with BioRender.com.

Most biological effects of dioxins are mediated by the aryl hydrocarbon receptor (AhR), a member of the nuclear receptor family (43) (Fig. 3*A*). The toxicity of dioxins depends on their affinity for AhR, with TCDD being the most potent AhR agonist. As such, the toxicity of dioxins and dioxin-like chemicals is quantified relative to TCDD using toxic equivalency (TEQ). Although TCDD is now highly regulated and has been largely phased out, it is still used as a model chemical to study the biological effects of other PCDD/Fs and dioxin-like chemicals.

The function of AhR has been extensively studied in tissues such as the liver and has been reviewed elsewhere (44, 45). In brief, the canonical genomic AhR pathway leads to the upregulation of AhR target genes, including xenobiotic metabolism genes, cytochrome *P*-450 (e.g., *Cyp1a1*), and inflammatory genes (e.g., *Il-6*, *Il-22*, *Lxa4*) (44); although essential, overactivation of this pathway can also generate reactive oxygen species (ROS) (46) (Fig. 3*A*). AhR also has noncanonical roles such as inhibiting cell cycle progression (via repression of S-phase genes), promoting inflammatory responses (via activation of NF-κβ), and promoting apoptosis. However, AhR has also been found to promote cell survival and proliferation, suggesting that the function of AhR varies in different cell types (44, 45, 47, 48). Finally, AhR also has antiestrogenic effects by promoting degradation of the estrogen receptors (ERs) (49) (Fig. 3*A*). It is therefore plausible that dioxins impair β-cell function and/or survival through AhR-mediated changes in Ca^2+^ and/or estrogen receptor signaling (Fig. 3*A*).

The general population is continuously exposed to low doses of PCDD/Fs mainly via the diet (39), but high-dose exposure is also common in occupational workers (50) and victims of chemical disasters (51). For example, occupational workers were estimated to have a mean PCDD/F serum concentration of 141 pg/g lipid (52), and populations exposed in PCDD/F-contaminated areas had serum concentrations in the range of 5–115 pg TEQ/g lipid (53–55). In contrast, a mean serum PCDD/F concentration of 7–14 pg TEQ/g lipid was reported in the United States (56) and Canadian (57) general populations.

### Epidemiological Evidence Linking Dioxins with T2D and β-Cell Dysfunction

Populations with high-level PCDD exposure consistently show positive associations between serum PCDD levels and T2D incidence (58–65), insulin resistance (66, 67), hyperglycemia (60, 64, 65, 68), metabolic syndrome (69), and increased T2D mortality risk (51, 70). In the general population, serum PCDD levels have also been associated with increased T2D risk (16, 71–74), although null associations for T2D (75, 76), insulin resistance (77), and metabolic syndrome (78) have been reported. Plasma insulin data are limited but show that populations exposed to high-dose PCDD have increased fasting and glucose-induced serum insulin levels (79–81). Taken together, epidemiological evidence suggests an increased risk of impaired glucose homeostasis in individuals exposed acutely to high doses of PCDDs, but this relationship is less consistent in the general population (Fig. 2*A*). These data also point to dioxin-induced β-cell dysfunction.

### Glucose Homeostasis and Plasma Insulin

**Figure 4.**
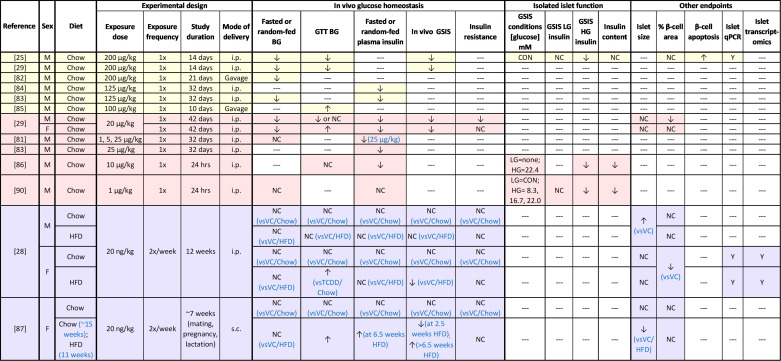
Summary of in vivo rodent studies with dioxin. Supraphysiological high-dose exposure studies are highlighted in yellow; physiological high-dose exposure studies are highlighted in pink; low-dose exposure studies are highlighted in purple. ↑, Increase; ↓, decrease; NC, no change; ‐‐‐, not measured. BG, blood glucose; CON, conventional glucose-stimulated insulin secretion (GSIS) conditions (LG = 2.8 mM, HG = 16.7 mM); GTT, glucose tolerance test; HFD, high-fat diet; i.p., intraperitoneal; s.c., subcutaneous; VC, vehicle control.

In line with epidemiological studies, high-dose dioxin exposure impaired glucose homeostasis in rodents (Fig. 4). Supraphysiological high-dose TCDD exposure generally caused prolonged hypoglycemia (25, 29, 82, 83) and hypoinsulinemia (25, 29, 83, 84) in male rodents, with the exception of one study that reported slight hyperglycemia (85). These findings suggest that acute dioxin exposure impairs insulin secretion; however, these models display significant weight loss, confounding interpretation of glucose and insulin data (25, 29, 82, 83, 85).

Male (29, 86) and female (29) mice exposed to a physiologically relevant high dose of TCDD maintained healthy body weight but also showed prolonged fasting hypoglycemia and hypoinsulinemia. There were also sex-specific effects of TCDD on glucose homeostasis (29, 86). TCDD-exposed males were hypoglycemic or normoglycemic during glucose tolerance tests (GTTs) and had increased insulin sensitivity during an insulin tolerance test (ITT) (29, 86), whereas TCDD-exposed females showed transient glucose intolerance and no change in insulin sensitivity compared to controls (29). These data suggest that females are more susceptible to TCDD-induced hyperglycemia than males, although more studies are needed.

Kurita et al. (86) reported that high-dose TCDD exposure did not reduce plasma insulin levels in male mice with a global AhR deletion, supporting a role for AhR in mediating the metabolic effects of TCDD in vivo. A β-cell-specific AhR-knockout model would be useful to investigate the role of AhR in mediating the effects of TCDD on β-cells specifically. It would also be interesting to investigate whether the sex-specific effects of TCDD involve interaction between AhR and estrogen receptors.

There are limited data regarding the metabolic effects of physiologically relevant low-dose TCDD exposure in vivo. Our laboratory found that prolonged low-dose TCDD exposure did not impact fasting blood glucose levels, glucose tolerance, insulin sensitivity, or plasma insulin levels in chow-fed male or female mice (28). However, when combined with high-fat diet (HFD) feeding, TCDD exposure accelerated the onset of HFD-induced glucose intolerance in female but not male mice (28). TCDD-exposed female mice also lacked the HFD-induced compensatory hyperinsulinemic response during a glucose challenge that was observed in HFD-fed vehicle-exposed mice. Impaired metabolic adaptability to HFD feeding following TCDD exposure in female mice was also seen in a related pregnancy model (87).

Collectively, in vivo dioxin data (Fig. 4) align with epidemiological studies showing that high-dose dioxin exposure impairs glucose homeostasis, although specific effects on glycemia and plasma insulin differed between humans and rodents. This discrepancy may be due to confounding variables in epidemiological studies. It is also possible that structural differences between human and rodent AhR may contribute to these differences. Human AhR has a 10-fold lower affinity for TCDD compared with mouse AhR (88), suggesting that dioxin exposure in rodents will have more drastic effects on glucose homeostasis compared with humans. Rodent studies can still be used to infer metabolic consequences of long-term dioxin exposure; our review suggests that TCDD decreases insulin secretion and increases susceptibility to hyperglycemia and diabetes, at least in females. Low-dose exposure to dioxin alone has minimal effects on glucose homeostasis but appears to induce maladaptive responses to HFD feeding in female mice. This may help explain the variable association between dioxin exposure and T2D risk in epidemiological studies of the general population; at low doses, the effect of dioxin on glucose homeostasis likely depends on other environmental T2D risk factors such as diet.

### β-Cell Function and Islet Biochemical Characterization

Our laboratory recently showed that in vitro TCDD exposure robustly induced CYP1A1 gene expression and enzyme activity in mouse and human islets (25), confirming activation of the AhR pathway in the endocrine pancreas. We further validated that TCDD activates AhR in islets in vivo. Systemic exposure to high (25, 29)- or low (25, 28)-dose TCDD in mice caused local upregulation of *Cyp1a1* in islets, and a single high dose of TCDD led to sustained upregulation of CYP1A1 enzyme activity in islets for at least 2 wk (25). In contrast, in immortalized β-cell lines, TCDD exposure modestly increased Cyp1a1 gene (25) and protein (89) expression but did not induce CYP1A1 enzyme activity (25), indicating that immortalized β-cell lines may not be the ideal model to study CYP-mediated effects of TCDD (or other AhR ligands); this is an important consideration when interpreting these data. However, studies in cell lines are still useful to study other AhR-mediated mechanisms in β-cells.

**Figure 5.**
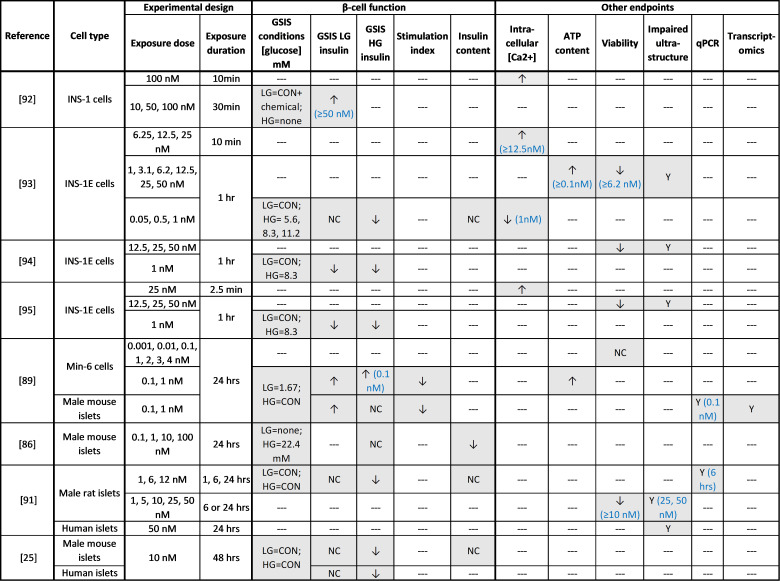
Summary of in vitro studies with dioxin. ↑, Increase; ↓, decrease; NC, no change; ‐‐‐, not measured. CON, conventional glucose-stimulated insulin secretion (GSIS) conditions (LG = 2.8 mM, HG = 16.7 mM).

Functional islet assessments after in vivo or in vitro TCDD exposure indicate that TCDD disrupts β-cell function (Figs. 4 and 5). High-dose TCDD exposure in vivo caused persistent β-cell dysfunction in isolated islets (25, 86, 90), including diminished GSIS (25, 86, 90), impaired insulin secretion in response to a nonglucose secretagogue, α-ketoisocaproate (90), and decreased insulin content (86, 90); this aligns with the reported hypoinsulinemia in TCDD-exposed rodents (25, 29, 81, 83, 84, 86). Likewise, in vitro high-dose TCDD exposure impaired GSIS in primary rodent and human islets but did not impact basal insulin secretion, insulin content, or *Ins1* gene expression (25, 91). One study in rodent islets reported no change in GSIS after TCDD exposure (86), although insulin content was reduced. In addition, high-dose TCDD exposure in immortalized β-cells increased basal insulin (92); GSIS was not assessed, making it difficult to interpret the data.

To our knowledge, no studies have assessed β-cell function in isolated islets ex vivo after low-dose TCDD exposure in vivo (Fig. 4); this is an important gap in the literature that should be addressed. The reported effects of low-dose TCDD exposure in vitro on insulin secretion vary but generally point to impaired GSIS. TCDD decreased basal insulin secretion and/or GSIS in isolated islets (91) and immortalized cell lines (93–95). One study reported increased insulin secretion under high-glucose conditions in TCDD-exposed islets, but the stimulation index (i.e., ratio of insulin release after high-glucose relative to low-glucose conditions) was reduced (89), indicating impaired β-cell function. Another study found decreased insulin content in TCDD-exposed rodent islets cells but no change in insulin secretion under high-glucose conditions; basal insulin secretion was not reported, so the stimulation index could not be determined (86). The variability between these studies may be due to differences in the exposure and/or GSIS protocols used (see Fig. 5). Regardless, these data imply that TCDD can directly impair β-cell function.

Although direct assessments of islet function ex vivo after in vivo TCDD exposure are lacking, transcriptomic analysis on isolated islets and pancreas histology from female mice exposed to prolonged low-dose TCDD ± HFD (28, 87) suggest that TCDD disrupts β-cell health and promotes maladaptive metabolic responses to HFD feeding. Numerous endocrine and metabolic pathways, including amino acid metabolism and circadian rhythm pathways, were uniquely enriched after TCDD-HFD coexposure compared with either TCDD or HFD exposure alone (28). Aberrant amino acid metabolism and circadian rhythm are both linked to metabolic dysfunction (96–99) and impaired GSIS (100–102). We also found that key markers of β-cell function and identity, such as MafA and Slc2a2, were reduced in TCDD-HFD islets compared to TCDD-Chow (28, 87). Finally, TCDD-HFD females displayed an increase in cytoplasmic proinsulin accumulation in β-cells, indicative of defective insulin processing and β-cell function (87).

These findings are largely supported by studies in isolated islets showing decreased expression of glucose homeostasis genes *Slc2a2* and *Gck* as well as key β-cell transcription factors *MafA, Pdx1,* and *FoxO1* following high-dose TCDD exposure in vitro (91). Transcriptomic analysis of male mouse islets after low-dose TCDD exposure in vitro revealed a total of 5,484 upregulated and 305 downregulated genes compared with DMSO exposure (89); several pathways involved in islet function and insulin secretion were altered, including phosphatidylinositol signaling system, Ca^2+^ signaling, mTOR signaling, Wnt signaling, insulin signaling, and type 2 diabetes mellitus (89). These findings suggest that TCDD exposure induces transcriptomic changes in β-cells that promote loss of β-cell identity and impaired metabolic adaptability, although further research is required to elucidate the underlying biochemical mechanisms involved.

There is strong biochemical evidence emerging that provides insight into how TCDD alters β-cell function (Fig. 5). TCDD exposure in immortalized β-cells increased ATP levels after a glucose stimulus (89) but had no effect on KCl-induced insulin secretion (93), suggesting a secretory defect linked to glucose sensing and/or mitochondrial function (Fig. 3*B*). Glucose-induced intracellular Ca^2+^ influx was also acutely abolished after TCDD exposure (93), indicating that TCDD directly modulates intracellular Ca^2+^ levels. Finally, suppression of GSIS by TCDD in human islets was prevented by cotreatment with a glucagon-like peptide-1 (GLP-1) receptor agonist, exendin-4 (25), which is known to potentiate GSIS and promote β-cell survival and proliferation in response to a metabolic stress (103, 104). These data suggest that either GLP-1 mitigates the toxic effects of TCDD on β-cells or TCDD impairs β-cell function through a GLP-1-dependent mechanism (Fig. 3*A*).

Coexposure of TCDD with an indirect AhR inhibitor (green tea polyphenol epigallocatechin 3-gallate, EGCG) rescued insulin suppression in immortalized β-cells (94), supporting a role of AhR in mediating the effects of TCDD on islets. Surprisingly, coexposure with cytokines completely prevented TCDD-mediated *CYP1A1* induction in human islets (25), suggesting an interaction between AhR and inflammation signaling pathways. This cross talk warrants further investigation to elucidate whether dioxin impacts β-cells differently under proinflammatory conditions such as obesity.

Taken together, both in vivo and in vitro studies point to suppressed insulin secretion in TCDD-exposed β-cells (Figs. 4 and 5), which supports the consistently reduced plasma insulin levels observed in TCDD-exposed rodents (25, 29, 81, 83, 84, 86) (Fig. 4). Detailed assessments of dynamic insulin secretion by perifusion in male and female islets would help clarify some of the discrepancies reported with static GSIS assays. There is also evidence that TCDD impairs mitochondrial function and Ca^2+^ influx, most likely through an AhR-dependent mechanism, which could impact both β-cell function and survival (Fig. 3*A*); direct measurements of mitochondrial function and energy metabolism in TCDD-exposed islets are needed. Additional studies in human islets from a diverse spectrum of donors are also needed since the magnitude of TCDD-induced insulin suppression varied among donors (25).

### **β**-Cell Stress and Viability

In vivo (Fig. 4) and in vitro (Fig. 5) studies suggest that the TCDD-induced suppression in insulin secretion is partially explained by β-cell loss. We reported a pronounced increase in β-cell apoptosis following supraphysiological high-dose TCDD exposure in male mice, although percent β-cell area per islet remained unchanged (25). Interestingly, the degree of β-cell apoptosis far exceeded that in either liver tissue or non-insulin^+^ pancreatic endocrine cells (25), suggesting that β-cells are particularly susceptible to dioxin-induced cytotoxicity. In line with these findings, male mice exposed to a physiological high dose of TCDD displayed a decrease in percent β-cell area per islet (29), likely a result of an earlier wave of β-cell death, although this remains to be confirmed. Surprisingly, female mice had normal islet endocrine composition despite developing transient glucose intolerance in vivo, suggesting a sex-dependent effect of TCDD on β-cell survival (29).

A decrease in β-cell viability was also observed after high-dose TCDD exposure in vitro (91, 93–95). Studies in both cell lines (92–95) and isolated islets (91) suggest that TCDD increases β-cell stress and apoptosis through Ca^2+^-dependent mitochondrial toxicity. TCDD caused mitochondrial and endoplasmic reticulum (ER) swelling, increased autophagic vesicles, altered mitochondrial membrane potential, altered expression of genes involved in ER stress (*Ire1*, *Chop*), and acutely increased intracellular Ca^2+^ concentration ([Ca^2+^]) (91–95); note that α-cell ultrastructure appeared normal after TCDD exposure in islets, further suggesting that β-cells are particularly susceptible to dioxin-induced cytotoxicity (91). Pretreatment with flunarizine, a T-type Ca^2+^ channel blocker, abrogated these effects (92), whereas coexposure of TCDD and either EGTA (a Ca^2+^ chelator) or dehydroascorbate (DHA, an antioxidant) prevented some but not all cell death. Preincubation with an AhR inhibitor, EGCG, also increased cell survival after high-dose TCDD exposure (93). Collectively, these data suggest that TCDD-induced cytotoxicity is AhR mediated and at least partially involves extracellular [Ca^2+^] and free radicals (93, 94).

In contrast to the high-dose TCDD models, low-dose TCDD does not appear to impair β-cell viability, at least not in the time frames reported to date. Prolonged low-dose TCDD exposure in vivo increased the average islet size in male mice and decreased percent β-cell area per islet in female mice (28). It remains unclear whether the decreased percent β-cell area in female mice is caused by increased β-cell apoptosis or another mechanism (e.g., β-cell dedifferentiation and/or decreased β-cell proliferation). In vitro low-dose TCDD exposure in cell lines (89) and isolated islets (91) had no effect on β-cell ultrastructure or apoptosis but increased expression of *iNos* (nitric oxide synthase) (91), which is associated with ER stress, inflammation, and β-cell dysfunction (105, 106). It is plausible that chronic iNos upregulation would eventually lead to apoptosis in a longer-term model of low-dose TCDD exposure.

### Summary of Dioxins and Future Perspectives

The in vivo and in vitro TCDD data align with epidemiological findings pointing to TCDD as causing β-cell toxicity (Fig. 2, Fig. 3*A*, Fig. 4, Fig. 5). Overall, systemic dioxin exposure led to impaired glucose homeostasis and altered insulin secretion, most likely due to impaired β-cell function and/or viability. High-dose TCDD exposure generally decreased insulin secretion by causing β-cell loss, potentially through a Ca^2+^-mediated mechanism. This is particularly concerning given the low regenerative capacity of β-cells. In contrast, low-dose TCDD exposure did not impact β-cell survival but rather impaired metabolic flexibility by altering metabolic pathways such as circadian rhythm and amino acid metabolism. We speculate that low-dose TCDD may also cause β-cell dedifferentiation, as indicated by loss of MAFA in insulin^+^ cells and suppressed GSIS, but additional validation is needed. There is compelling evidence that, regardless of dose, TCDD activates AhR signaling in islets, but whether AhR mediates the observed effects on β-cell function and health needs to be investigated using β-cell-specific AhR-knockout models.

Rodent studies consistently report sex-specific effects of TCDD on glucose homeostasis, β-cell survival, and responses to a secondary metabolic stressor in vivo. TCDD-exposed female mice show a greater risk of developing diabetes compared with male mice, especially when cotreated with HFD. These findings emphasize the importance of considering sex in studies investigating pollutant-induced diabetes. Although most epidemiological studies do not stratify data by sex, our meta-analysis also suggested that there are sex-specific associations between TCDD exposure and diabetes risk, with females having a higher risk than men in populations exposed to high-dose TCDD (107). Exposure to secondary metabolic stressors such as pregnancy or high-fat/high-calorie diets are also rarely considered in epidemiological studies. This may explain some of the variation in associations between dioxin exposure and T2D incidence in humans. Moreover, few epidemiological studies assess serum insulin or β-cell function (e.g., HOMA-β), meaning that potential associations with impaired insulin secretion are overlooked.

## POLYCHLORINATED BIPHENYLS

### Overview of Polychlorinated Biphenyls and Human Exposure

Polychlorinated biphenyls (PCBs, Fig. 1*B*) were widely used in commercial industries such as production of coolants and plasticizers before being banned in the United States in the 1970s (108, 109). There are 209 different PCB congeners, classified on the basis of their chemical structure as either non-ortho-substituted (coplanar and “dioxin-like,” DL) or ortho-substituted (noncoplanar and “non-dioxin-like,” NDL) (Fig. 1*B*). Of the 209 PCB congeners, only 12 are considered “dioxin-like” based on their affinity for AhR and mechanism of action similar to dioxins (110) (Fig. 3*A*). In contrast, studies in tissues including liver and muscle have shown that NDL-PCBs have different and more complex routes of toxicity, namely via the nuclear receptors PXR (pregnane X receptor) and CAR (constitutive aldosterone receptor) (Fig. 3*B*) (109, 111, 112). Similar to AhR, activation of PXR and CAR leads to the upregulation of xenobiotic metabolism enzymes (e.g., *Cyp3a* and *Cyp2b*), which can lead to ROS production. PXR and CAR activation can also alter various metabolic signaling pathways through interactions with peroxisome proliferator-activated receptors (PPARs), thyroid hormones, FOXO1, and glucocorticoid receptor (112–114). Some NDL-PCBs are also known to interact with ryanodine receptors (RyRs) (115), which play a crucial role in Ca^2+^ signaling (116). Therefore, it is plausible that both DL- and NDL-PCBs disrupt β-cell health and function but through different modes of action.

Human exposure to PCBs often occurs via complex mixtures known as Aroclor congeners (117). Although the production of PCBs and Aroclor congeners is now well regulated in many countries and has been largely phased out (118), humans are still exposed to PCBs through diet, consumer products, airborne particles, and dust (119). PCBs remain detectable in the general population worldwide, with an estimated serum half-life of 5–10 yr depending on the congener (120, 121). For example, median serum lipid content of the most common NDL-PCB congeners was between 23 and 162 ng/g in Europe (19, 122), the United States (123), and Canada (124, 125).

### Epidemiological Evidence Linking PCBs with T2D and β-Cell Dysfunction

A limited number of studies have reported a link between disaster-type and occupational exposure to PCBs and increased risk of prediabetes (126) and T2D (15, 127–129). In the general population, serum levels of PCBs are consistently associated with increased risk of prediabetes (130, 131), T2D (16, 18, 71, 72, 75, 76, 122–125, 130–143), and gestational diabetes (144) (Fig. 2*A*). Serum PCB concentrations have also been associated with other markers of dysglycemia including insulin resistance (77, 145–149), abnormal glucose tolerance (19), fasting hyperglycemia (130, 149, 150), and metabolic syndrome (151). Plasma insulin data are limited; however, high serum PCB levels are associated with fasting hypoinsulinemia (150) and decreased plasma insulin levels during a glucose challenge (17) and negatively correlated with HOMA-β (19, 148), suggesting a link between PCB exposure and impaired β-cell function.

### Glucose Homeostasis and Plasma Insulin

**Figure 6.**
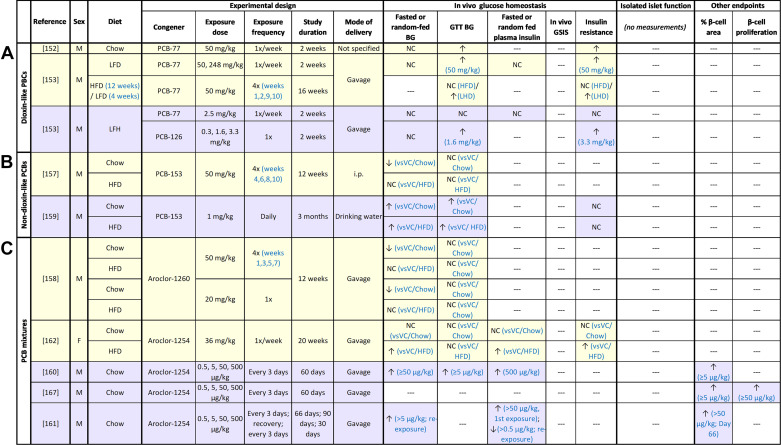
Summary of in vivo rodent studies with polychlorinated biphenyls (PCBs), including dioxin-like PCBs (*A*), non-dioxin-like PCBs (*B*), and PCB mixtures (*C*). Supraphysiological high-dose exposure studies are highlighted in yellow; physiological high-dose exposure studies are highlighted in pink; low-dose exposure studies are highlighted in purple. ↑, Increase; ↓, decrease; NC, no change; ‐‐‐, not measured. BG, blood glucose; GTT, glucose tolerance test; HFD, high-fat diet; i.p., intraperitoneal; LFD, low-fat diet; VC, vehicle control.

Consistent with human data, PCB exposure in rodents generally impaired glucose homeostasis in vivo, but very little data are available for plasma insulin levels (Fig. 6). Exposure to both high (152, 153)- and low(153)-dose DL-PCBs induced glucose intolerance and insulin resistance in chow- or low-fat diet (LFD)-fed male mice but did not impact fasting blood glucose or plasma insulin levels, indicating an increased susceptibility to developing diabetes. High-dose DL-PCB exposure had no impact on glucose homeostasis in HFD-fed male mice, but when subsequently switched to LFD, DL-PCB-exposed mice developed glucose intolerance and insulin resistance, suggesting an impaired ability to adapt to metabolic change (153). These findings also suggest that PCB exposure blunts the beneficial effects of weight loss on glucose homeostasis, potentially because of the release of PCBs from adipose tissue back into the circulation (154–156); whether PCBs released into the circulation are redistributed to other tissues such as the pancreas remains unclear. Interestingly, administration of an AhR antagonist prevented glucose intolerance in DL-PCB-exposed mice (152, 153), confirming that DL-PCBs exert their effects through AhR (Fig. 3*A*).

In contrast to DL-PCBs, high-dose exposure to NDL-PCBs (157) or NDL-PCB-rich Aroclors (158) consistently caused hypoglycemia in chow-fed male mice but had no effect on glucose tolerance, whereas low-dose exposure caused fasting hyperglycemia and glucose intolerance (159–161). Plasma insulin levels were not measured in high-dose exposure models, but two studies reported hyperinsulinemia following low-dose Aroclor exposure (160, 161) and then fasting hypoinsulinemia after recovery and subsequent reexposure to Aroclor (161). These data suggest that NDL-PCBs hyperactivate β-cells, which can lead to long-term impairment of β-cell function and hyperglycemia, especially when faced with a second hit of chemical exposure. Interestingly, low-dose but not high-dose exposure to NDL-PCBs (159) or Aroclor also exacerbated HFD-induced hyperglycemia in male mice, suggesting an interaction between NDL-PCBs and other T2D risk factors.

Unfortunately, data in female rodents are very limited. One study showed that supraphysiological high-dose Aroclor exposure did not impact glucose homeostasis in chow-fed female mice but exacerbated HFD-induced hyperglycemia, hyperinsulinemia, and insulin resistance (162). These findings indicate that the effects of Aroclor on glucose homeostasis are sex specific. Research in females should be prioritized to better understand this phenotype.

In summary, DL- and NDL-PCBs disrupt glucose homeostasis in male mice but likely through different mechanisms (Fig. 3, Fig. 6). Exposure to NDL-PCB Aroclor mixtures caused hyperglycemia and altered plasma insulin levels in both male and female rodents, although a secondary metabolic stressor was required in females. Interestingly, the metabolic effects of NDL-PCB-rich Aroclor mixtures resembled those of single NDL-PCBs, indicating that coexposure to different PCBs does not have an additive effect; additional studies with a more diverse range of Aroclors are needed to carefully compare different PCB mixtures, especially with physiologically relevant doses. In addition, detailed measurements of plasma insulin concentrations are needed to compare the effects of mixtures versus individual PCB congeners on β-cell function in vivo.

### β-Cell Function and Islet Biochemical Characterization

**Figure 7.**
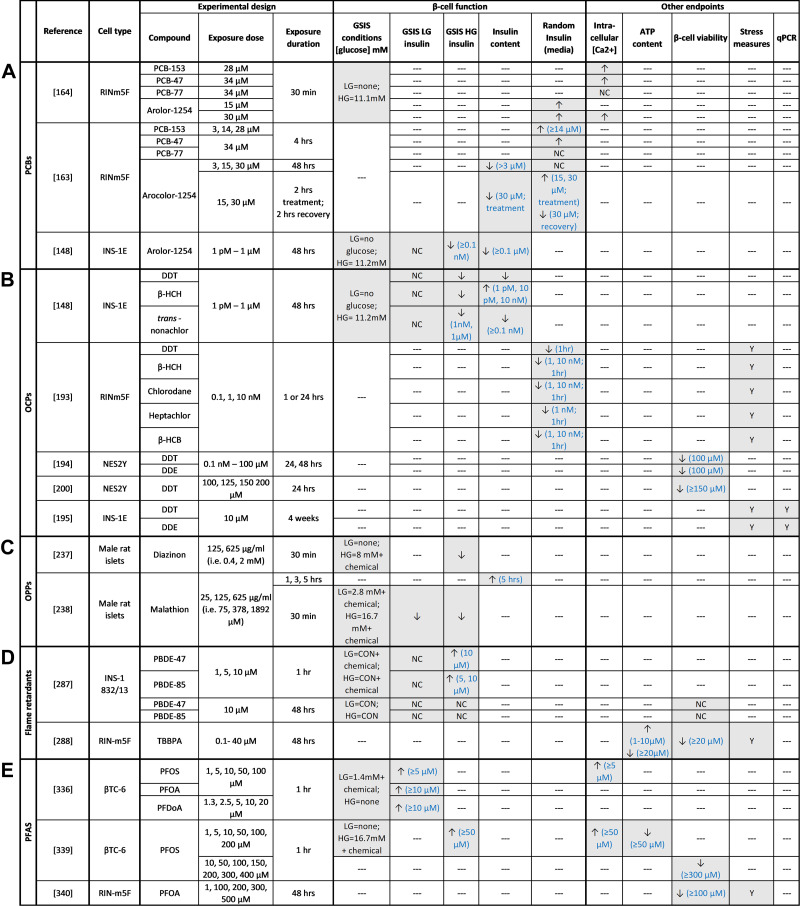
Summary of in vitro studies with polychlorinated biphenyls (PCBs; *A*), organochlorine pesticides (OCPs; *B*), organophosphate pesticides (OPPs; *C*), flame retardants (*D*), and per- and polyfluoroalkyl substances (PFAS; *E*). ↑, Increase; ↓, decrease; NC, no change; ‐‐‐, not measured. β-HCB, hexachlorobenzene; β-HCH, hexachlorocyclohexane; CON, conventional glucose-stimulated insulin secretion (GSIS) conditions (LG = 2.8 mM, HG = 16.7 mM). DDE, dichlorodiphenyldichloroethylene; DDT, dichlorodiphenyltrichloroethane; PBDE, polybrominated diphenyl ethers; PFDoA, Perfluorododecanoic acid; PFOA, perfluorooctanoic acid; PFOS, perfluorooctane sulfate; TBBPA, tetrabromobisphenol A.

There are limited studies examining the impact of direct PCB exposure on β-cells in vitro, and all were conducted in immortalized β-cell lines (Fig. 7*A*); collectively these studies point to PCB-induced β-cell dysfunction. High-dose Aroclor exposure acutely increased random insulin concentration in media but decreased insulin concentration after a recovery period (163); this is in line with in vivo data showing initial hyperinsulinemia followed by hypoinsulinemia after a recovery period and Aroclor reexposure (161). Longer-term Aroclor exposure also reduced GSIS and insulin content but did not alter basal insulin secretion in INS-1E cells (148); note that basal insulin levels were measured in the absence of glucose as opposed to a low glucose concentration. Assessment of high-dose exposure to individual DL- and NDL-PCBs showed that only NDL-PCBs increased insulin concentration in the media (163). These findings indicate that Aroclors and NDL-PCBs acutely hyperstimulate β-cells, leading to long-term susceptibility to β-cell dysfunction. This is in line with in vivo findings suggesting that single NDL-PCBs have an effect on β-cell function similar to NDL-PCB mixtures. However, assessments of GSIS using conventional methods are warranted to support these findings.

Interestingly, high-dose exposure to NDL-PCBs and Aroclors acutely increased intracellular Ca^2+^ influx (164), which could explain the hypersecretion response. Pretreatment with a Ca^2+^ channel blocker only partially attenuated the increased insulin secretion (164), suggesting that other mechanisms are also involved. In fact, the Aroclor-induced increase in insulin release was completely prevented by pretreatment with a Ca^2+^/calmodulin-dependent protein kinase II (CaM kinase II) inhibitor (164), indicating a role for this enzyme in mediating Ca^2+^-dependent signal transduction in NDL-PCB-exposed β-cells. DL-PCBs had no effect on intracellular Ca^2+^ levels, which confirms that DL-PCBs and NDL-PCBs likely work through different mechanisms.

Although in vitro cell line studies report PCB-induced changes in insulin release (Fig. 7*A*), most were conducted in RINm5F cells, which are known to be deficient in glucose-regulated insulin secretion (165, 166) and thus are limited as a model of human β-cells. Future studies should confirm these findings in primary rodent and human islets.

### **β**-Cell Stress and Viability

Studies assessing the effects of PCB exposure on β-cell stress and viability are limited. Only one research group assessed islet cell proliferation following prolonged low-dose Aroclor exposure in male mice in vivo; they found increased β-cell area and proliferation and decreased α-cell area but no change in α-cell proliferation (Fig. 6) (160, 161, 167). These data are consistent with the hyperinsulinemia observed in vivo (160–162), but whether this increase in β-cell proliferation is an adaptive response to β-cell loss and/or dysfunction remains unclear. Future research should directly assess β-cell viability after in vivo exposure to PCBs and Aroclor mixtures in both male and female mice, as well as after in vitro exposure to islets.

### Summary of PCBs and Future Perspectives

In sum, both the in vivo and in vitro data support the epidemiological literature describing associations between PCB exposure and increased diabetes risk (Fig. 2); however, additional research is required to understand the direct impact of PCBs on β-cells, including GSIS, Ca^2+^ flux, β-cell survival, and gene expression (Fig. 3). Interestingly, both DL-PCBs and NDL-PCBs impaired glucose homeostasis in vivo (Fig. 6), but only NDL-PCBs appear to directly alter β-cell function in immortalized cell lines in vitro (Fig. 7*A*). These findings suggest that NDL-PCBs and NDL-PCB-rich Aroclor mixtures are more of a concern than DL-PCBs, although more detailed assessments in primary islets are needed. Future studies should prioritize high-throughput screening methods to highlight specific congeners or mixtures of concern. There is also a clear absence of work in female rodents. Studies should prioritize assessing the metabolic effects of PCBs in both sexes.

## ORGANOCHLORINE PESTICIDES

### Overview of Organochlorine Pesticides and Human Exposure

Organochlorine pesticides (OCPs) are a class of POPs that include compounds such as dichlorodiphenyltrichloroethane (DDT), dichlorodiphenyldichloroethylene (DDE; a metabolite of DDT), endosulfan, hexachlorobenzene (HCB), and hexachlorocyclohexane (HCH), among many others (168) (Fig. 1*C*). The widespread use of OCPs in the previous century resulted in expansive environmental contamination (168). Although many OCPs were phased out in the 1970s, they continue to be used in developing nations (169), leading to persistent human exposure, mainly via diet. Recent studies report median serum lipid concentrations of OCPs in the range of 10–536 ng/g in Asian, South African, European, and Canadian populations (123, 170–172).

The mechanism of action for OCPs has mainly been studied in the liver and nervous system. Similar to NDL-PCBs, OCPs upregulate CYP enzymes via CAR activation (173). OCP exposure also leads to increased oxidative stress, mitochondrial dysfunction, and apoptosis in neuronal cells in vitro (174). Finally, OCPs are well known for interfering with neural activity by inhibiting GABAergic actions and ion flux in the brain, thereby causing continuous neuron depolarization (168, 174, 175). Given that β-cells also express GABA (gamma-aminobutyric acid) receptors and GABA activation stimulates insulin release and β-cell regeneration and inhibits apoptosis (176), it is plausible that OCPs could have similar effects on β-cell function and health (Fig. 3*B*).

### Epidemiological Evidence Linking OCPs with T2D and **β**-Cell Dysfunction

A limited number of studies have reported a link between occupational exposure to OCPs and increased T2D risk (177–179). There are more studies in the general population, and these consistently report associations between elevated serum OCP levels and increased risk of prediabetes (126, 130, 131) and T2D (16, 18, 19, 71, 75, 76, 78, 122–125, 130, 131, 133, 134, 136–140, 143, 180–188) (Fig. 2*A*). OCPs are also positively associated with markers of impaired glucose homeostasis including insulin resistance (145–148), glucose intolerance (19, 128), and fasting hyperglycemia (78, 124, 130, 137, 149). Importantly, there is some evidence that serum OCPs are linked to β-cell dysfunction in humans, including a significant association with decreased glucose-stimulated (17, 148) and fasted (128, 135) plasma insulin and decreased HOMA-β (19, 148).

### Glucose Homeostasis and Plasma Insulin

**Figure 8.**
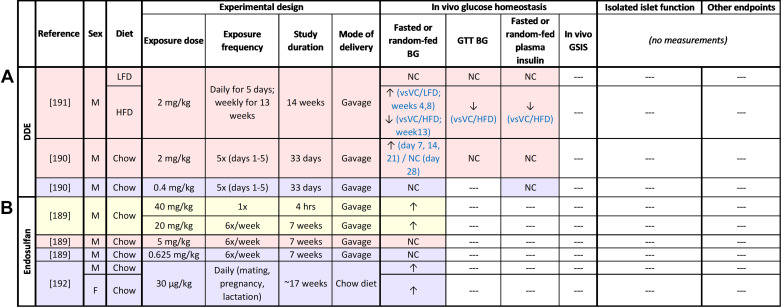
Summary of in vivo rodent studies with organochlorine pesticides (OCPs), including dichlorodiphenyldichloroethylene (DDE; *A*) and endosulfan (*B*). Supraphysiological high-dose exposure studies are highlighted in yellow; physiological high-dose exposure studies are highlighted in pink; low-dose exposure studies are highlighted in purple. ↑, Increase; ↓, decrease; NC, no change; ‐‐‐, not measured. BG, blood glucose; GTT, glucose tolerance test; HFD, high-fat diet; LFD, low-fat diet; VC, vehicle control.

Rodent studies with OCPs are limited to DDE and endosulfan, both of which cause impaired glucose homeostasis in vivo (Fig. 8). Short-term exposure to supraphysiological or physiological high-dose endosulfan (189) or DDE (190) caused transient hyperglycemia in male mice but had no effect on fasting insulin levels or glucose tolerance. Chronic exposure to physiologically relevant high-dose DDE had no effect on fasting glycemia or glucose tolerance in LFD-fed males (191), but the first metabolic assessment was 4 wk after DDE exposure started, a time point at which DDE-induced hyperglycemia had resolved in another study (190). These studies suggest that the effects of high-dose OCP on glucose homeostasis are transient. However, when combined with HFD feeding, chronic high-dose DDE exposure had more persistent effects on glucose homeostasis. DDE exacerbated HFD-induced fasting hyperglycemia and markedly suppressed HFD-induced fasting hyperinsulinemia (191), suggestive of impaired β-cell adaptation to HFD feeding. Glucose-stimulated plasma insulin levels were not measured in this study (191), making it difficult to assess the effects of OCPs on β-cell function in vivo.

Prolonged exposure (5–7 wk) to low-dose DDE (190) or endosulfan (189) had no effect on fasting blood glucose (189, 190) or plasma insulin levels (190) in male mice, whereas chronic exposure (17 wk) caused fasting hyperglycemia in both males and females (192). The effect of chronic low-dose OCP exposure on basal or glucose-stimulated plasma insulin levels was not reported.

In summary, both the in vivo and epidemiological data suggest that OCPs cause hyperglycemia (Fig. 2), but more studies are needed to investigate the impact of OCPs on plasma insulin levels (Fig. 8). Interestingly, one study reported suppressed HFD-induced hyperinsulinemia in DDE-exposed male mice (191), which is reminiscent of our findings with TCDD-exposed female mice (28). However, this study did not assess the effect of DDE in HFD-fed females, and only one study looked at OCPs in chow-fed female mice. Thus, future in vivo work should include both sexes and consider the effect of OCPs with and without a secondary metabolic stressor. Rodent studies on OCPs other than DDE and endosulfan are also needed.

### **β**-Cell Function and Islet Biochemical Characterization

Studies assessing the effects of OCPs on β-cell function in vitro point to impaired insulin secretion but are limited to immortalized β-cell lines (Fig. 7*B*). High-dose exposure to various OCPs (chlordane, heptachlor, DDT, β-HCH, or β-HCB) rapidly decreased insulin concentration in media, an effect that resolved over time (193); however, GSIS was not assessed in this study, making it difficult to interpret the extent to which insulin secretion was affected. Another study showed that acute OCP (β-HCH, *trans*-nonachlor, or DDT) exposure dose-dependently decreased GSIS but did not alter basal insulin secretion, although basal measurements were taken in glucose-free KRBB rather than the conventional 2.8 mM low-glucose condition (148). Interestingly, DDT and *trans*-nonachlor also decreased insulin content, whereas β-HCH either increased or had no effect on insulin content, depending on the dose (148), suggesting that the mechanism by which OCPs impact β-cell function varies between compounds.

Studies assessing OCP-induced molecular changes in β-cell lines are also limited. Prolonged low-dose DDT or DDE exposure reduced protein expression of several cytoskeletal proteins (194, 195) and decreased the glycolytic enzyme α-enolase (194), as shown by two-dimensional (2D) gel electrophoresis. Upregulation of vitamin D binding protein (VDBP) following DDT or DDE exposure was also observed (195), which is interesting because VDBP polymorphisms have been associated with T2D risk and VDBP autoantigenicity is linked to type 1 diabetes (T1D) risk (196–199). Finally, DDT and DDE altered intracellular insulin levels, including reduced proinsulin, insulin monomer, and gene expression of *Ins1* and *Ins2* (195). This is consistent with the impaired insulin secretion observed after in vivo (191) and in vitro (148, 193) OCP exposure and suggests that OCPs impair β-cell function, perhaps in part by repressing insulin gene expression and downregulating cytoarchitecture necessary for insulin vesicle exocytosis. Whether these effects are mediated by GABA receptors remains to be examined.

### **β**-Cell Stress and Viability

Studies assessing the effects of OCPs on β-cell viability are limited to immortalized β-cell lines and show evidence of OCP-induced β-cell stress and decrease viability (Fig. 7*B*). Exposure to acute high-dose DDT and DDE significantly decreased cell viability (194, 200) and increased markers of apoptosis (200). 2D gel electrophoresis also showed that prolonged high-dose DDT exposure upregulated several ER stress proteins, a mitochondrial chaperone protein, and proteins involved in cell morphology maintenance while downregulating proteins related to mitochondria, cytoskeleton, transcription and translation, oxidative stress response, and heat shock (195). Acute low-dose OCP exposure had no effect on viability (194) but increased ROS production (193).

These data suggest that high-dose OCP exposure acutely activates a wide variety of stress response pathways while inhibiting essential cell maintenance pathways, leading to β-cell death. Although low-lose OCP exposure did not cause obvious β-cell death in these models, the observed increase in ROS could eventually lead to β-cell death with longer-term exposure.

### Summary of OCPs and Future Perspectives

Collectively, the in vivo and in vitro data suggest that OCP exposure suppresses insulin secretion and promotes hyperglycemia, perhaps in part by altering β-cell function and promoting β-cell death, although the specific mechanism of action remains unclear (Fig. 2, Fig. 3*B*, Fig. 7*B*, and Fig. 8). Follow-up studies in primary rodent or human islets are needed to better understand the impact of OCPs on β-cell function and stress pathways. The use of more physiologically relevant concentrations of OCPs in both males and females should also be prioritized. Finally, given the widespread use of a variety of OCPs in agriculture, future investigations might consider evaluating different OCP mixtures that reflect current environmental contamination.

## ORGANOPHOSPHATE PESTICIDES

### Overview of Organophosphate Pesticides and Human Exposure

Organophosphate pesticides (OPPs) are widely used phosphorus-containing acid derivatives that include compounds such as diazinon, dichlorvos, dimethoate, monocrotophos, and malathion (201) (Fig. 1*D*). OPPs have largely replaced OCPs in industry since the 1970s, although given associations between OPPs and adverse health outcomes the manufacture of some OPPs has also been regulated, phased out, or banned globally (202). However, some OPPs such as malathion remain poorly regulated (203).

Humans are primarily exposed to OPPs via diet (204). Exposure to low doses of OPPs, as in the general population, is difficult to quantify because of the short half-life (<24 h) of many OPPs in human serum (205); however, there is some evidence that serum OPP concentrations are declining in the general population since the implementation of regulatory actions (206). Human exposure to OPPs is usually determined based on the concentration of OPP metabolites in urine, which ranges between 0.3 and 2.2 ng/mL in the Canadian population (207). Mean serum levels in the United States, Greece, and Asia range between 0.2 and 9.3 mg/mL (208).

OPPs primarily exert toxic effects through inhibition of acetylcholinesterase (AChE), which can result in acute toxicity due to acetylcholine (ACh) accumulation and cholinergic stress. The conventional function of AChE is to hydrolyze ACh and terminate impulse transmissions at cholinergic synapses (202, 209). However, ACh is also critical for β-cell function and stimulates GSIS via both parasympathetic innervation of islets (210) and local production by α-cells (211). AChE is expressed in islets, and AChE inhibition acutely increased insulin secretion in human islets (209, 211). Therefore, it is plausible that OPPs could have profound effects on insulin secretion via direct inhibition of AChE in islets (Fig. 3*B*).

### Epidemiological Evidence Linking OPPs with T2D and β-Cell Dysfunction

Epidemiological studies examining OPP exposure in the general population are lacking; however, there is mounting evidence linking occupational exposure to OPPs with increased T2D risk (177, 178), fasting hyperglycemia (212), insulin resistance (212), and gestational diabetes (213). We only found one study that measured plasma insulin levels and found pronounced hyperinsulinemia in malathion-exposed farmers (212). In fact, fasting insulin levels were more than twice as high in malathion-exposed farmers compared with control subjects (212), suggestive of OPP-induced β-cell defects.

### Glucose Homeostasis and Plasma Insulin

**Figure 9.**
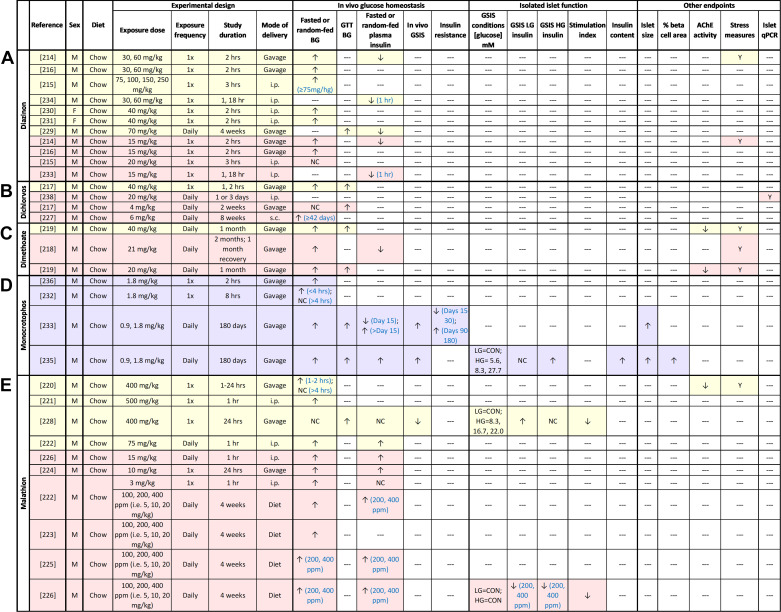
Summary of in vivo rodent studies with organophosphate pesticides (OPPs), including diazinon (*A*), dichlorvos (*B*), dimethoate (*C*), monocrotophos (*D*), and malathion (*E*). Supraphysiological high-dose exposure studies are highlighted in yellow; physiological high-dose exposure studies are highlighted in pink; low-dose exposure studies are highlighted in purple. ↑, Increase; ↓, decrease; NC, no change; ‐‐‐, not measured. BG, blood glucose; CON, conventional glucose-stimulated insulin secretion (GSIS) conditions (LG = 2.8 mM, HG = 16.7 mM); GTT, glucose tolerance test; i.p., intraperitoneal; s.c., sub-cutaneous.

Consistent with epidemiological studies, in vivo OPP exposure caused hyperglycemia in rodents, but data on plasma insulin levels were variable (Fig. 9). Numerous studies found that supraphysiological and physiological high-dose diazinon (214–216), dichlorvos (217), dimethoate (218, 219), and malathion (220–226) caused prolonged hyperglycemia (214–216, 218–227) and glucose intolerance (217, 219, 228, 229) in male rodents. Males exposed to high-dose dimethoate remained hyperglycemic and hypoinsulinemic even after a recovery period, suggesting long-term effects of transient high-dose OPP exposure (218). Female mice exposed to high-dose diazinon also developed hyperglycemia (230, 231), although more studies in females are needed. Similar to high-dose OPP exposure, low-dose monocrotophos exposure caused hyperglycemia (232, 233) and glucose intolerance (233) in male rats, although the degree of hyperglycemia varied over time (233).

There is compelling evidence that OPP-induced hyperglycemia is linked with impaired β-cell function (Fig. 9). High-dose exposure to diazinon (214, 229, 234) or dimethoate (218) decreased plasma insulin (214, 218, 229, 233, 234) and increased C-peptide levels (234) in male rodents, pointing to a deficiency in proinsulin processing or abnormally rapid insulin clearance. In contrast, high-dose exposure to malathion consistently increased plasma insulin levels (222, 224–226); glucose-induced plasma insulin levels were not assessed in these studies. Whether these differences are related to the specific OPP used or variation in the animal models (i.e., duration and mode of exposure) is unclear. Low-dose exposure to monocrotophos also impaired plasma insulin levels, initially causing fasting hypoinsulinemia followed by pronounced fasting hyperinsulinemia and increased glucose-induced plasma insulin levels (233, 235). Hyperinsulinemia in this study preceded the onset of insulin resistance (233), pointing to a β-cell-driven effect of low-dose monocrotophos.

There are numerous proposed mechanisms for how OPPs disrupt plasma insulin and glucose levels. Interestingly, the effect of diazinon on blood glucose varied depending on the time of administration, with blood glucose levels being higher after diurnal exposure (215). These findings suggest that OPPs disrupts glucose homeostasis by altering circadian rhythms. Another study showed that coadministration of diazinon with phosphodiesterase inhibitors partially restored plasma insulin levels and fully normalized blood glucose levels (214), indicating that the effects of OPPs are mediated, at least in part, by intracellular second messengers cAMP and cGMP (Fig. 3*B*). Finally, pretreatment with ACh receptor antagonists (232), as well as α-adrenergic and β-adrenergic receptor antagonists (236), prevented monocrotophos-induced fasting hyperglycemia. The authors suggested that monocrotophos increases blood glucose levels by promoting gluconeogenesis through both parasympathetic and sympathetic mechanisms. Whether exposure to monocrotophos, and other OPPs, alters islet function through these neural mechanisms remains unclear.

Collectively, the in vivo data convincingly show that OPPs induce hyperglycemia (Fig. 9), which supports the epidemiological literature (Fig. 2*A*). However, the effects on fasting and glucose-induced plasma insulin levels were variable, suggesting that the effect of OPPs on β-cell function differs depending on the compound and/or exposure model used (Fig. 9). Future studies should provide a more thorough assessment of plasma insulin in vivo, including GSIS. Coexposure of rodents to OPPs along with other metabolic stressors should also be prioritized, as studies to date are limited to only chow-fed rodents. Additionally, all in vivo studies with OPPs in the past 30 years have been in male rodents; investigations into sex-specific effects of OPPs are urgently needed. Finally, the majority of OPP studies were conducted with supraphysiological or disaster/occupational-type high-dose exposure protocols. Future studies should assess the effects of low-dose OPP exposure.

### **β**-Cell Function and Islet Biochemical Characterization

Studies assessing the effect of in vivo OPP exposure on β-cell function in isolated islets are limited and show variable results but collectively point to reduced insulin secretion (Fig. 9). Acute high-dose malathion administration in vivo significantly increased basal insulin but did not impact GSIS, resulting in a suppressed stimulation index ex vivo (228) (Fig. 9*E*). In contrast, prolonged high-dose malathion decreased both basal insulin and GSIS (226); although not calculated, the stimulation index was also clearly suppressed (226). Interestingly, KCl-stimulated insulin secretion was not affected by *in vivo* malathion exposure (226), pointing to a defect in glucose sensing and/or mitochondrial function. The suppressed insulin secretion ex vivo is counterintuitive given the profoundly increased plasma insulin levels observed in malathion-exposed rats (226).

In vitro exposure to high-dose diazinon (237) or malathion (238) in isolated male rat islets also significantly reduced insulin secretion (237, 238) but increased insulin and C-peptide content (238) (Fig. 7*C*). Direct malathion exposure not only completely abolished GSIS but actually inversed insulin secretion such that basal release was higher than glucose-stimulated release (note that vehicle-exposed islets displayed the expected 2-fold stimulation index) (238). Collectively, these findings suggest that exposure to malathion, and most likely other OPPs, increases basal insulin secretion to the point of β-cell exhaustion, preventing a proper response to a glucose stimulus; more detailed assessments of β-cell function after OPP exposure are warranted to support these findings.

In contrast to high-dose exposure models, male rats chronically exposed to low-dose monocrotophos in vivo displayed normal basal insulin secretion, increased GSIS, and increased insulin content in isolated islets (235), which is consistent with the pronounced hyperinsulinemia observed in vivo (233, 235) (Fig. 9*D*). Since this increase in GSIS could eventually lead to β-cell exhaustion (239), it is plausible that both high- and low-dose OPP exposure have the potential to reduce insulin secretion.

While studies measuring insulin secretion are limited, several studies assessed islet biochemistry and suggest impaired β-cell function. In vivo exposure to diazinon (234), malathion (222, 228), and monocrotophos (235) all significantly increased activity of glucokinase (GCK) (222, 235) and Krebs cycle enzymes (222, 234, 235), as well as the concentration of key metabolites, such as pyruvate, malate, and ATP (235), in isolated islets. These findings are somewhat surprising given that diazinon and malathion/monocrotophos had opposite effects on plasma insulin in vivo. More research is clearly required to elucidate the role of GCK and Krebs cycle enzymes in mediating the effects of OPPs on β-cell function. Interestingly, in vivo dichlorvos did not alter these markers (240), indicating that different OPPs work through different mechanisms of action.

OPPs may also exert their effects on β-cells by altering pancreas/islet composition. High-dose exposure to dimethoate in vivo increased pancreas mass (219) and reduced the number of insulin granules within β-cells (218), which could contribute to the hypoinsulinemia observed in vivo (218, 219). However, these were qualitative observations of electron microscopic (EM) images, and quantitative analysis is required to validate these findings. In contrast, male rats chronically exposed to low-dose monocrotophos showed increased islet area and insulin content in whole pancreas (233, 235), which corresponds with the hyperinsulinemia observed in vivo (233, 235) (Fig. 9*D*).

Finally, high-dose exposure to dimethoate (219) and malathion (220) in vivo transiently decreased activity of pancreatic AChE, implicating a role for AChE in mediating the effects of OPPs on islets (Fig. 3*B*). Co-administration of diazinon with ACh and a cholinesterase inhibitor (neostigmine) in vitro partially prevented the observed diazinon-induced decrease in insulin secretion (237) (Fig. 7*C*). Coadministration of diazinon with an antioxidant (α-tocopherol) in vivo completely prevented the decrease in insulin secretion (237), whereas coadministration with a muscarinic receptor antagonist (atropine), Ca^2+^ channel blocker (nifedipine), α-adrenergic receptor antagonist (phenoxybenzamine), or β-adrenergic receptor antagonist (propranolol) did not improve insulin secretion (237) (Fig. 7*C*). Therefore, OPPs likely act on islets through a combination of free radical-induced stress and cholinergic-dependent parasympathetic mechanisms.

In summary, despite the wealth of data on the in vivo effects of OPPs on glucose homeostasis and plasma insulin (Fig. 9), there are comparatively few studies that describe the effects of OPP exposure on β-cell function or islet biochemistry (Fig. 7*C* and Fig. 9). However, the available data suggest that OPPs impair β-cell function. Detailed analysis of dynamic insulin secretion following both in vivo and in vitro OPP exposure would help to better elucidate the effects of OPPs on islet function. Future studies should also access mitochondrial function in OPP-exposed islets.

### **β**-Cell Stress and Viability

Studies assessing β-cell stress following OPP exposure are also limited and have only been conducted after in vivo exposure, not in vitro exposure (Fig. 9). High-dose diazinon exposure in vivo acutely increased nitric oxide concentration, a lipid peroxidation marker (thiobarbituric acid reactive substances, TBARS), and TNF-α protein in isolated islets (214). Likewise, prolonged exposure to dimethoate increased pancreatic ROS levels and activity of numerous antioxidant enzymes [superoxide dismutase (SOD), catalase (CAT), glutathione peroxidase (GPX), glutathione-*S*-transferase (GST)] (219). Dimethoate altered β-cell ultrastructure including ER dilation and vacuolization (218), although these were entirely qualitative observations. Finally, high-dose malathion administration in vivo led to increased lipid peroxidation (TBARS) in whole pancreas (220) and various indicators of oxidative stress in isolated islets, including increased ROS production, lipid peroxidation, 8-oxo-2′-deoxyguanosine (8-OHdG, an oxidative stress marker), and protein carbonyl levels (228). Taken together, these findings suggest that OPPs cause β-cell stress and alter normal cellular signaling, but additional studies are needed, particularly to assess β-cell viability after OPP exposure.

### Summary of OPPs and Future Perspectives

There is convincing evidence at all levels linking OPP exposure to β-cell dysfunction (Fig. 2, Fig. 7*C*, and Fig. 9). In vivo rodent exposure to diazinon, dichlorvos, dimethoate, and malathion consistently led to hyperglycemia in rodent studies (Fig. 9). Additionally, short-term OPP exposure decreased plasma insulin levels in nearly all rodent studies, whereas long-term exposure to either monocrotophos or malathion led to hyperinsulinemia in vivo (Fig. 9). This is consistent with malathion-exposed farmers having pronounced hyperinsulinemia compared with the control population (212).

Pronounced AChE inhibition was observed in the pancreas of dimethoate- and malathion-exposed rats, indicating that these OPPs (and presumably others) likely act on the pancreatic cholinergic pathway (Fig. 3*B*). AChE inhibition should prevent local breakdown of ACh, which is a known stimulator of insulin secretion in mouse and human β-cells via the muscarinic ACh receptors (210, 241). Therefore, local AChE inhibition in the pancreas could explain the hyperinsulinemia in long-term malathion exposure studies. However, dimethoate exposure caused hypoinsulinemia (218), suggesting that other pathways are likely also playing a role.

Across all OPP studies, there is a clear absence of work in female rodents to investigate potential sex differences. Additionally, markers of β-cell dysfunction such as the proinsulin-to-insulin ratio, more thorough histological characterization of islet endocrine cell distribution and composition, and transcriptomic analysis of islets after in vivo OPP exposure would provide important insight into the pancreatic islet response to OPPs.

These data also raise potential concerns about the current level of human exposure to OPPs. The acceptable daily intake of malathion for humans was established as 0.3 mg/kg. This was set to be 100-fold lower than the no-observed-adverse-effect level (NOAEL) for malathion, which was established as 29 mg/kg/day based on a 2-yr toxicity and carcinogenicity study in rats (222, 242). However, the studies reviewed here clearly show adverse effects of malathion and other OPPs on glucose homeostasis and insulin secretion at doses much lower than the previously established NOAEL and suggest that this value should be reevaluated.

## FLAME RETARDANTS

### Overview of Flame Retardants and Human Exposure

There are four classes of flame retardants: halogenated, organophosphorus (OPFRs), inorganic, and nitrogen-containing flame retardants (243) (Fig. 1*E*). Halogenated flame retardants and OPFRs are persistent and are thus the focus of this review. Halogenated flame retardants include brominated flame retardants (BFRs) and chlorinated flame retardants (CFRs). BFRs are historically the most widely used class of flame retardants; the main commercial BFRs include polybrominated diphenyl ethers (PBDEs), polybrominated biphenyls (PBBs), hexabromocyclododecane (HBCD), and tetrabromobisphenol A (TBBPA) (243, 244). BFRs are commonly added in industrial and commercial items such as clothing and children’s toys to increase thermal stability (244, 245), leading to widespread environmental contamination (243, 246). Most BFRs have a half-life ranging from 2 to 29 yr (247, 248), although some have a relatively short half-life of <100 days (249, 250). Since 2009, several BFRs have been listed as POPs by the Stockholm Convention (251) and are now well regulated (251). However, humans are still continuously exposed to BFRs through air, diet, dust, consumer items, and occupationally (252, 253). Recent studies have reported mean PBDE serum lipid concentrations of 2–25 ng/g in United States (254, 255) and Canadian (172, 254) populations.

As a result of emerging health concerns (256), BFRs have largely been replaced by CFRs (e.g., dechlorane plus, DP, Fig. 1*E*) and OPFRs in industry (257, 258). Despite their recent emergence, median serum lipid concentrations of CFRs have been reported in the range of 42–456 ng/g in Chinese occupational workers (259–261), and 0.11–13.7 ng/g in the Canadian (262) and Chinese (260, 263) general populations.

OPFRs are organic esters of phosphoric acid that are widely used as flame retardants in commercial products (e.g., furniture, textiles, and electronics), as plasticizers in consumer products, and in construction materials (257). OPFR compounds have a relatively short half-life (∼4–50 days) compared with BFRs (264) but are still widely detected in humans. Mean concentrations of OPFR urinary metabolites have been reported within the range of 0.038–4 ng/mL in the United States (265–267), Canadian (268), and Chinese (269) general populations; urinary metabolite concentrations offer a more reliable measurement of OPFR levels in humans compared with serum levels.

The mechanism through which flame retardants exert cellular toxicity has not been completely elucidated, but inhibition of thyroid hormone signaling is one proposed mode of action for BFRs (270). Given that thyroid hormones are essential in maintaining β-cell function and MAFA expression (271), it is possible that flame retardants could impair insulin secretion by disrupting thyroid hormone signaling in islets (Fig. 3*B*).

### Epidemiological Evidence Linking Flame Retardants with T2D and β-Cell Dysfunction

To our knowledge, no epidemiological studies have examined the link between high-dose exposure to flame retardants and increased diabetes risk or impaired glucose homeostasis. However, several studies have shown that firefighters have increased risk of diabetes (272–274); whether this is associated with high exposure to flame retardants is unclear.

In the general population, the data linking flame retardants to increased T2D risk are variable. Serum PBDEs (275, 276) and PBBs (138), as well as dietary exposure to HBCD and PBDE (277), have been positively associated with increased T2D risk. Serum BFRs have also been associated with markers of impaired glucose homeostasis including fasting hyperglycemia (135), metabolic syndrome (275), and insulin resistance (278). However, other studies reported no association between BFR exposure and T2D risk (76, 140–142). There are limited data on plasma insulin levels in humans exposed to BFRs; in a Cree population without diabetes, there was no association between serum PBDE and plasma insulin levels but HOMA-β was negatively correlated with plasma PBDE levels, suggesting a possible link to β-cell dysfunction (135). No epidemiological studies have assessed a link between CFRs and diabetes risk.

Urinary metabolites of OPFRs have also been associated with increased T2D incidence (279), hyperglycemia, metabolic syndrome (280), and prediabetes (231). Interestingly, urinary OPFR metabolites were also inversely associated with fasting insulin in both female and male adolescents (281). Given the increase in OPFR use, ongoing analyses of the possible association between OPFR exposure and T2D should be prioritized.

### Glucose Homeostasis and Plasma Insulin

**Figure 10.**
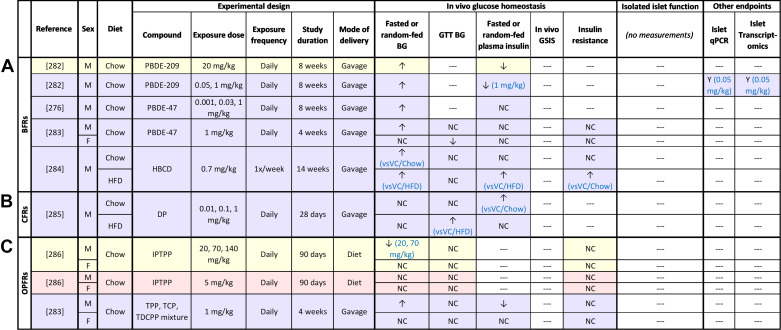
Summary of in vivo rodent studies with flame retardants, including brominated flame retardants (BFRs; *A*), chlorinated flame retardants (CFRs; *B*), and organophosphorus flame retardants (OPFRs; *C*). Supraphysiological high-dose exposure studies are highlighted in yellow; physiological high-dose exposure studies are highlighted in pink; low-dose exposure studies are highlighted in purple. ↑, Increase; ↓, decrease; NC, no change; ‐‐‐, not measured. BG, blood glucose; DP, dechlorane plus; GTT, glucose tolerance test; HBCD, hexabromocyclododecane; HFD, high-fat diet; IPTPP, isopropylated triphenyl phosphate; TCP, tricresyl phosphate; TDCPP, Tris (1,3-dichloro-2-propyl)phosphate; TPP, triphenyl phosphate; VC, vehicle control.

Collectively, in vivo data show that BFRs impair glucose homeostasis in rodents (Fig. 10*A*). Exposure to either supraphysiological high- or low-dose PBDEs (276, 282, 283) or HBCD (284) consistently led to fasting hyperglycemia in chow-fed male rodents (276, 282–284) but no change in glucose tolerance or insulin sensitivity (283, 284). Plasma insulin data were more variable but generally point to hypoinsulinemia (282) or no change in plasma insulin levels (276, 283) in chow-fed males. This variability seems to be BFR dependent, suggesting that different BFRs alter glucose homeostasis via different mechanisms. In contrast, female rodents exposed to low-dose PBDE had normal fasting glycemia and plasma insulin and slightly improved glucose tolerance (283). Data on glucose-induced plasma insulin levels are lacking in all these studies and would help clarify discrepancies in random-fed plasma insulin data. In a HFD model, HBCD exposure worsened HFD-induced hyperglycemia, random-fed hyperinsulinemia, and insulin resistance in male rodents (284), indicating that BFRs impair adaptability to a metabolic stressor.

Only one study assessed the metabolic effects of in vivo CFR exposure (Fig. 10*B*). Prolonged low-dose DP exposure substantially increased random-fed plasma insulin levels in chow-fed but not HFD-fed male mice (285). Interestingly though, DP exposure exacerbated HFD-induced glucose intolerance yet had no impact on glycemia in chow-fed mice (285). Note that GSIS was not assessed in vivo or in isolated islets ex vivo, making it difficult to determine whether DP alters β-cell function.

The effects of high-dose OPFRs differed from both BFRs and CFRs (Fig. 10*C*). Chronic high-dose OPFR exposure caused fasting hypoglycemia in male rats without altering glucose tolerance or insulin sensitivity (286); unfortunately, this study did not assess plasma insulin levels. Low-dose OPFR exposure led to fasting hyperglycemia and decreased random-fed plasma insulin levels in male mice (283) and had no effect on overall glucose tolerance in either sex (283). Therefore, the adverse metabolic consequences of low-dose replacement flame retardants resemble those of phased-out BFRs, although more detail on the effects of OPFRs on glucose homeostasis and β-cell function is needed. High-dose (286) and low-dose (283) OPFR exposure had no effect on glucose homeostasis in females, further suggesting that males are more susceptible to impaired glucose homeostasis following exposure to flame retardants.

### β-Cell Function and Islet Biochemical Characterization

Islets were not isolated from any of the in vivo flame retardant studies (Fig. 10). However, a qualitative assessment of hematoxylin and eosin (H&E)-stained pancreas from PBDE-exposed rats showed no obvious morphological changes (276). Clearly, more work is needed to investigate the effects of flame retardants on islets in vivo.

Likewise, there are limited data available on the impact of flame retardants on β-cells in vitro, and all studies were conducted in immortalized cell lines (Fig. 7*D*). Acute high-dose exposure to PBDEs under high-glucose conditions rapidly increased insulin secretion, but this effect was not seen after longer exposure in culture media (287). It is possible that PBDEs acutely stimulate insulin secretion and these effects resolve over time, but more research using conventional GSIS conditions is needed. Interestingly, the increase in insulin secretion following PBDE exposure was further amplified by coexposure with thyroid hormone (T_3_) and prevented by cotreatment with a thyroid hormone receptor antagonist (287), suggesting that flame retardants mediate their effects on β-cell function by altering thyroid signaling (Fig. 3*B*). Another study showed that acute exposure to low-dose BFR increased ATP levels but high-dose BFR decreased ATP levels (288), indicating that flame retardants may disrupt β-cell mitochondrial function. Assessing the impact of other flame retardants, including newer CFRs and OPFRs, on GSIS and mitochondrial function in isolated islets should be prioritized.

### β-Cell Stress and Viability

We found one study in immortalized cells showing that acute high-dose TBBPA dramatically decreased cell viability and increased apoptosis, cytochrome *c* release, nitric oxide levels, and ROS levels compared with vehicle exposure (288) (Fig. 7*D*). Validation in isolated islets is required.

### Summary of Flame Retardants and Future Perspectives

Taken together, flame retardants consistently alter glucose homeostasis in rodent models, which supports the associations between flame retardants and diabetes reported in the epidemiological literature (Fig. 2 and Fig. 10). The insulin data were more variable or lacking entirely but overall showed a decrease or no change to basal plasma insulin levels following exposure to flame retardants, except for two studies that showed hyperinsulinemia in HFD-fed mice (Fig. 10); these findings suggest an interactive effect between flame retardants and HFD feeding on plasma insulin levels. Note that none of the rodent studies with flame retardants measured glucose-induced plasma insulin levels in vivo or GSIS in isolated islets ex vivo. Future studies should prioritize measuring plasma insulin in more than just random-fed conditions, and β-cell function should ideally be assessed in isolated islets, along with other measures of islet health. Furthermore, given the sex differences reported in both BFR- and OPFR-exposed rodents, additional studies involving both sexes are warranted.

Finally, in vitro studies on the direct effects of flame retardants on insulin secretion should include primary rodent and human islets and expand beyond BFRs to also consider the newer CFR and OPFR replacement flame retardants. The potential role of thyroid hormone receptors in mediating effects of BFRs on insulin secretion also warrants further investigation.

## PER- AND POLYFLUOROALKYL SUBSTANCES

### Overview of Per- and Polyfluoroalkyl Substances and Human Exposure

Per- and polyfluoroalkyl substances (collectively termed PFAS, Fig. 1*F*) are amphipathic compounds (289) commonly used as surfactants, polymers, and firefighting foams; PFAS are found in consumer items such as nonstick pans (e.g., Teflon) and clothing (e.g., Gore-Tex) (290). Examples of widely used PFAS include perfluorooctanoic acid (PFOA) and perfluorooctane sulfonate (PFOS, Fig. 1*F*). Importantly, the Stockholm Convention added PFOS and PFOA to the list of POPs in 2009 and 2016, respectively (291).

There is some regulatory action on manufacturing and/or safety limits of these longer-carbon chain PFAS in the United States (292) and the European Union (293), which has led to the increased manufacturing and use of shorter-chain moieties. However, biomonitoring studies continue to report that the primary legacy PFAS (PFOA, PFOS) are still detectable in the majority of the population (294–297). Mean serum concentrations of PFOA in Canada (207), the United States (298, 299) and Europe (300, 301) ranged from 1.3 to 5.3 ng/mL, whereas PFOS levels ranged from 3.4 to 52.0 ng/mL. Half-lives of long-chain PFAS isomers are reported to be 2.7–15.5 yr (302–305), whereas shorter-chain PFAS have half-lives ranging from 44 days to 1.5 yr (294). Thus, the scientific community has called for increased regulation, toxicological testing, and limited use of PFAS (306).

Most adverse outcomes of PFAS exposure are associated with increased oxidative stress and subsequent mitochondrial dysfunction and apoptosis (307). PFAS interact with various nuclear receptors, including PPARs, ERs, and PXR in tissues such as muscle (307, 308); however, the main mechanism reported is PPAR activation (307). This is interesting given that PPARs promote β-cell function and survival (309–311); PPARs potentiate insulin secretion and reduce cellular stress caused by ROS (312). However, long-term activation of PPARs in β-cells following PFAS exposure could eventually be detrimental, and it is also possible that PFAS could act on β-cells through PPAR-independent mechanisms (Fig. 3*B*).

### Epidemiological Evidence Linking PFAS with T2D and β-Cell Dysfunction

Individuals occupationally exposed to PFAS have higher diabetes-related mortality than reference populations (313–316). Likewise, in the general population serum PFAS levels are associated with increased risk of T2D (299, 317–322), prediabetes (317), and T2D-related mortality (323). Serum PFAS have also been associated with markers of impaired glucose homeostasis including insulin resistance (299, 324), glucose intolerance (321), increased fasting blood glucose (299, 325), and metabolic syndrome (326). However, other studies report no association between serum PFAS and T2D risk (327–329), fasting blood glucose (318, 327, 328, 330), or metabolic syndrome (330) and inverse associations with T1D risk (331), T2D risk (321, 331, 332), glucose intolerance (321), and fasting blood glucose (326).

There is also evidence that PFAS exposure may be linked to β-cell dysfunction in humans. Serum PFAS levels were positively associated with increased fasting insulin (299, 324) and proinsulin (299), increased insulin during a GTT (299), and decreased proinsulin-to-insulin ratio (319) and HOMA-β (299, 324, 325). However, studies have also reported no link between PFAS and fasting insulin (318, 322, 330). Although the data are variable, PFAS should be considered a priority for further exploring links to adverse metabolic outcomes and β-cell health.

### Glucose Homeostasis and Plasma Insulin

**Figure 11.**
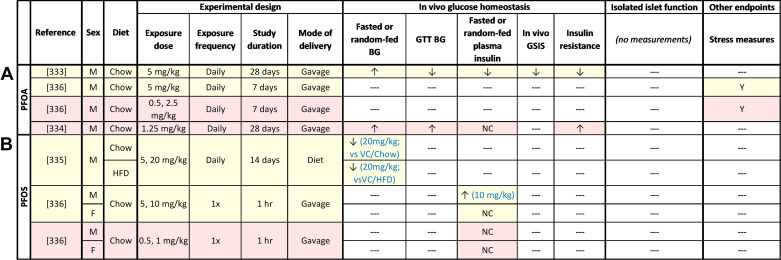
Summary of in vivo rodent studies with per- and polyfluoroalkyl substances (PFAS), including perfluorooctanoic acid (PFOA; *A*) and perfluorooctane sulfate (PFOS; *B*). Supraphysiological high-dose exposure studies are highlighted in yellow; physiological high-dose exposure studies are highlighted in pink. ↑, Increase; ↓, decrease; NC, no change; ‐‐‐, not measured. BG, blood glucose; GTT, glucose tolerance test; HFD, high-fat diet; VC, vehicle control.

Only two studies examined the impact of PFOA on glucose homeostasis in vivo, reporting largely opposite effects (Fig. 11*A*). Male mice exposed to prolonged supraphysiological high-dose PFOA had decreased glucose-induced plasma insulin and increased fasting glucose but improved glucose tolerance and increased insulin sensitivity compared with control animals (333). In contrast, male mice exposed to a physiologically relevant high dose of PFOA had increased fasting glucose levels, mild glucose intolerance, and reduced insulin sensitivity but normal fasting insulin (334); glucose-stimulated plasma insulin levels were not measured in this study. Therefore, high-dose PFOA consistently causes fasting hyperglycemia, but more studies are needed to resolve the variability in glucose tolerance and insulin secretion results (Fig. 11*A*).

In contrast to PFOA, supraphysiological high-dose PFOS exposure caused hypoglycemia in chow- and HFD-fed male mice (335) (Fig. 11*B*). Acute supraphysiological, but not physiological, high-dose PFOS exposure also increased serum insulin levels in male but not female mice (336). However, it is difficult to make conclusions about the effects of in vivo PFOS exposure on β-cell function since no study reported long-term effects of PFOS on fasted or glucose-stimulated plasma insulin levels. Interestingly, in mice with a global GPR40 (free fatty acid receptor 1) knockout, serum insulin levels remained unchanged after PFOS exposure (336), suggesting a role for GPR40 in mediating the effects of PFOS on insulin secretion (Fig. 3*B*). GPR40 is a G protein-coupled receptor that potentiates GSIS following fatty acid stimulation, but overactivation of GPR40 is linked to impaired β-cell function (337). GPR40 also interacts with PPARs (338); whether GPR40 activation mediates the metabolic effects of in vivo PFAS via PPARs requires further investigation. Future studies using a β-cell-specific GPR40-knockout model would also be of interest to further examine the mechanism of PFOS action in vivo.

### β-Cell Function and Islet Biochemical Characterization

Studies assessing β-cell function after PFAS exposure are limited to immortalized cell lines (Fig. 7*E*) and generally point to increased insulin secretion, which is consistent with the in vivo data showing PFOS-induced hyperinsulinemia in mice (336). Acute (1 hour) high-dose exposure to PFOS (336, 339), PFOA (336), or perfluorododecanoic acid (PFDoA) (336) in low (336)- or high (339)-glucose conditions stimulated insulin release. This is concerning since chronic increase in insulin can lead to reduced β-cell function long term (239). PFOS also rapidly increased intracellular Ca^2+^ levels (336, 339) and decreased the ATP-to-ADP ratio (339), suggesting that PFAS impair β-cell function by modulating Ca^2+^ flux and mitochondrial function.

Cotreatment with a GPR40 antagonist in vitro prevented the PFOS-induced increase in insulin secretion and intracellular Ca^2+^ (336, 339); this is in line with in vivo data in GPR40-knockout mice (336). In contrast, the effects of PFOS were not prevented by coadministration of a PPARγ antagonist but were partially attenuated by blocking downstream targets of GPR40 (phospholipase C and L-type Ca^2+^ channels) (339), implying a role for GPR40 that is independent of PPARs (Fig. 3*B*).

### **β**-Cell Stress and Viability

Studies assessing PFAS-induced stress in β-cells or islets are limited (Fig. 7*E* and Fig. 11). Prolonged supraphysiological PFOA exposure in vivo caused a dose-dependent increase in a marker of lipid peroxidation [8-iso-prostaglandin 2α (8-iso-PGF2α)] and gene expression of antioxidant enzymes in whole pancreas (336). Whether these findings translate to islets requires further investigation. Studies in immortalized β-cell lines also point to impaired β-cell viability following PFAS exposure; acute exposure to high-dose PFOS decreased viability (339, 340) and increased apoptosis, ROS levels, nitric oxide levels, TNF-α levels, and cytochrome *c* release (340). Assessment of β-cell stress and viability after low-dose PFAS exposure is needed.

### Summary of PFAS and Future Perspectives

Taken together, these data suggest that long-chain PFAS acutely stimulate insulin secretion (Fig. 2, Fig. 7*E*, and Fig. 11), possibly through activation of GPR40 (Fig. 3*B*), which could eventually lead to β-cell exhaustion. However, there remain substantial gaps in the literature about the impact of PFAS on β-cells. For example, in vivo studies assessing long-term metabolic outcomes, including plasma insulin levels, using physiologically relevant PFAS dosing protocols are needed in both male and female rodents. Future studies should also examine the effect of PFAS on GSIS in vitro, including both low and high glucose concentrations within the same study and ideally via perifusion of primary islets. Importantly, the concentrations of PFOS used in in vitro studies were only slightly below the concentrations shown to induce cytotoxicity (100–500 μM) in β-cell lines (339, 340). Studies in primary rodent and human pancreatic islets using lower PFAS concentrations would be ideal to expand on the work in immortalized β-cell lines.

We did not find any rodent studies that examined the metabolic impact of other long-chain PFAS [e.g., perfluorohexane sulfonate (PFHxS), PFDoA] or the newer short-chain replacement PFAS. Studies looking at the impact of short-chain PFAS on glucose homeostasis and β-cell function in vivo and in vitro should be prioritized to compare the effects of replacement chemicals with legacy PFAS.

## DISCUSSION AND CONCLUSIONS

The widespread use of POPs in agricultural and manufacturing industries has led to global environmental contamination. Humans are exposed to complex chemical mixtures through diet, air particles, or household and commercial products; mixtures of POPs are consistently found in detectable concentrations within human tissues, including the pancreas (23, 24). Our review provides an in-depth summary of the mounting evidence implicating POPs in dysfunctional glucose homeostasis, changes in β-cell function, and altered metabolic and oxidative stress pathways in islets (Figs. 2–11).

Supraphysiological and physiological high-dose exposure to most POPs led to hyperglycemia in male rodents, except for dioxins and PFOS, which triggered hypoglycemia. In vivo plasma insulin data are lacking or limited for several classes of POPs, but the available data from studies with high-dose dioxins, OPPs, and flame retardants consistently show reduced plasma insulin levels following chemical exposure. These findings are largely supported by ex vivo and in vitro islet studies showing reduced GSIS after high-dose dioxin, PCB, OCP, and OPP exposure; insufficient data are available to make conclusions about the effect of flame retardants or PFAS on β-cell function. Taken together, these data suggest that acute exposure to POPs promotes hyperglycemia and reduces insulin secretion, which likely increases diabetes risk; however, high-dose in vivo studies have limited applicability to the general population.

Studies assessing the effect of longer-term low-dose POP exposure are limited, but collectively the data with PCBs, OCPs, OPPs, and flame retardants show hyperglycemia in chow-fed male rodents; there are limited data on the effects of these POPs on plasma insulin levels or GSIS ex vivo, but the available data point to impaired β-cell function. The effect of low-dose OPPs was particularly clear, causing hyperglycemia, glucose intolerance, increased plasma insulin levels, and/or insulin resistance in males; unfortunately, there were no studies on low-dose OPPs in female rodents to compare. Regardless, OPPs should be considered priority chemicals in toxicological assessment and pollutant regulation.

High-dose and low-dose POP exposure consistently exacerbated HFD-induced hyperglycemia and impaired fasting and/or glucose-induced plasma insulin levels in both male and female rodents. Data from TCDD and PCB studies show that this effect is more pronounced and consistent in HFD-fed females than males. HFD studies with OCP, OPP, flame retardant, and PFAS exposure in females are lacking. Overall, the available data suggest that POP exposure impairs metabolic adaptability to other environmental stressors, which is concerning given the worldwide increase in poor diet and sedentary lifestyle. These findings emphasize the need to study the interaction between POP exposure and other metabolic stressors on glucose homeostasis and β-cell function.

The mechanisms by which POPs impair glucose homeostasis and β-cell function/health remain unclear; however, molecular-level data point to defects in mitochondrial function and Ca^2+^ influx (Fig. 3). Exposure to TCDD, OPPs, flame retardants, and PFAS consistently altered intracellular ATP levels in β-cells/islets, and OPP exposure altered expression of key metabolic enzymes and intermediate metabolites; therefore, POPs may disrupt glucose metabolism in β-cells, which could directly impair GSIS. In addition, TCDD, PCBs, and PFAS generally increased Ca^2+^ influx. Given the importance of Ca^2+^ in maintaining β-cell function, survival, and regenerative capacity (341), dysregulated Ca^2+^ homeostasis could be a key mechanism by which POPs exert β-cell toxicity. Future research should provide more detailed assessments of mitochondrial function and Ca^2+^ mobilization after POP exposure. Finally, RNA sequencing (RNAseq) and histological analysis of TCDD-exposed islets point to β-cell dedifferentiation as another potential mechanism for POP-induced β-cell dysfunction. The data to support this hypothesis are compelling but limited and so far restricted to dioxins. Future studies should prioritize assessing epigenetic, transcriptomic, and proteomic changes associated with loss of β-cell identity following in vivo and in vitro POP exposure. Current data suggest that POPs cause these molecular-level changes through different pathways. For example, dioxins and DL-PCBs may exert their effects through the AhR pathway, OPPs through the ACh pathway, flame retardants (specifically BFRs) through the thyroid hormone receptor, and PFAS through GPR40 (Fig. 3); studies in β-cell-specific knockout models for these pathways are warranted.

Although available studies strongly suggest that POPs increase diabetes risk, at least in part by driving β-cell defects, there are important gaps in the literature that need to be addressed. First, rodent studies on dioxins, OPPs, BFRs, and OPFRs all demonstrated sex-specific effects on glucose homeostasis, yet most studies only used male mice. Given that females seem more susceptible to impaired metabolic adaptability than males after exposure to dioxins and PCBs, future studies should prioritize assessing sex differences. Second, many rodent studies lacked a complete assessment of islet function, morphology, or endocrine composition. If plasma insulin levels were reported in vivo, most studies only reported random-fed or fasting insulin at a single time point. Very few studies isolated islets for ex vivo functional studies or transcriptomics analysis to better understand islet- and β-cell-specific effects of POP exposure. We also recommend thorough characterization of islet histology, such as β-cell mass, β-cell apoptosis/proliferation, and islet size in the pancreas of POP-exposed rodents.

Prioritizing environmental contaminants of interest will continue to be a significant challenge in the field. Many in vitro studies currently focus on exposure to a single POP in an immortalized β-cell line. Since humans are more frequently exposed to POP mixtures, rather than specific compounds in isolation, protocols that assess multiple pollutants simultaneously in a high-throughput manner will prove useful. It would also be ideal for in vitro studies to prioritize contaminants of interest using either primary islets or stem cell-derived pancreatic endocrine cells as a screening platform; the benefit of using stem cells in high-throughput screening has been reviewed elsewhere (37). This approach was used successfully by Zhou and colleagues (38) to screen the ∼2,000 compounds from the Phase I Toxicity Forecaster (ToxCast) library provided by the US Environmental Protection Agency (EPA) in human stem cell-derived β-like cells. Importantly, they identified an insecticide, propargite, that decreased survival of human β-like cells in vitro, an effect that translated into a mouse model in vivo (38). This elegant study focused on viability of insulin^+^ cells as the main end point for screening, but future studies could also consider GSIS as an outcome measure to identify contaminants that impact β-cell function.

In conclusion, all POPs assessed in this review show associations with increased diabetes risk. Although the endocrine pancreas is not typically considered a target organ for pollutant-induced adverse health outcomes, our review provides extensive evidence that POPs directly impair β-cell function and health (Figs. 2–11). These findings emphasize the need to investigate the impact of pollutants on both glucose homeostasis and β-cells directly in toxicology assessments and implementation of pollutant regulations.

## GRANTS

This research was supported by a Canadian Institutes of Health Research (CIHR) Project Grant (no. PJT-2018-159590) and a Natural Sciences and Engineering Research Council of Canada (NSERC) Discovery Grant (RGPIN-2017-06272). M.P.H. was supported by a CIHR CGS-D award.

## DISCLOSURES

No conflicts of interest, financial or otherwise, are declared by the authors.

## AUTHOR CONTRIBUTIONS

M.P.H. and J.E.B. prepared figures; M.P.H., G.M., and J.E.B. drafted manuscript; M.P.H., G.M., E.M.M., I.P., and J.E.B. edited and revised manuscript; M.P.H., G.M., E.M.M., I.P., and J.E.B. approved final version of manuscript.
